# Recent Advancements in Tin Halide Perovskite-Based Solar Cells and Thermoelectric Devices

**DOI:** 10.3390/nano12224055

**Published:** 2022-11-17

**Authors:** Ajay Kumar Baranwal, Shuzi Hayase

**Affiliations:** Info-Powered Energy System Research Center, The University of Electro-Communications, Tokyo 182-8585, Japan

**Keywords:** tin halide, perovskite, solar cells, thermoelectric devices

## Abstract

The excellent optoelectronic properties of tin halide perovskites (Sn-PVKs) have made them a promising candidate for replacing toxic Pb counterparts. Concurrently, their enormous potential in photon harvesting and thermoelectricity applications has attracted increasing attention. The optoelectronic properties of Sn-PVKs are governed by the flexible nature of SnI_6_ octahedra, and they exhibit extremely low thermal conductivity. Due to these diverse applications, this review first analyzes the structural properties, optoelectronic properties, defect physics, and thermoelectric properties of Sn-PVKs. Then, recent techniques developed to solve limitations with Sn-PVK-based devices to improve their photoelectric and thermoelectric performance are discussed in detail. Finally, the challenges and prospects for further development of Sn-PVK-based devices are discussed.

## 1. Introduction

Lead halide perovskite (Pb-PVK) has emerged as a versatile semiconducting material with extensive applications in photovoltaics [1], thermoelectricity [2], light-emitting diodes [3], laser detectors [4], piezoelectricity [5], radiation detectors [6], and optical fibers [7]. Pb-PVKs have a 3D crystal structure with ABX_3_ formulae, where A is an organic or inorganic monoatomic element (methyl ammonium (CH_3_NH_3_/MA), formamidine (CH_3_NH_2_NH/FA), or Cesium/Cs), B is a bivalent metal ion (Pb^2+^, Sn^2+^, or Ge^2+^), and X is a halide ion (I^−^, Br^−^, or Cl^−^). The B metal ion of the PVK crystal is surrounded by corner-dividing BX_6_ octahedra, which are flexible enough to allow such diverse electronic applications. PVK semiconductor thin films can be coated at low temperatures using a solution printing method, which makes the entire fabrication process economical and promising for mass production. The excellent optical and electronic properties of Pb-PVK have made possible a jump in the photoconversion efficiency of solar cells from an initial 3.1% to a record-breaking 25.7% within a short time of research [8,9], surpassing crystalline indium phosphide crystalline solar cells (24.20%), cadmium-free copper indium gallium selenide solar cells (23.35%), and close to crystalline Si solar cells (26.70%) [10], because of the exceptional optical-electrical properties of ambipolar, high defect tolerance, small exciton binding energy, long carrier diffusion length, and high absorption coefficient [11,12,13]. However, despite bearing such promising optoelectronic properties, the notorious toxicity of Pb has raised concerns about commercial implementations. Consequently, various studies have attempted to substitute Pb with other abundant, robust, and biocompatible metals, such as Germanium (Ge), Antimony (Sb), Bismuth (Bi), Titanium (Ti), Copper (Cu), and Tin (Sn) [14,15,16]. Replacing Pb with other metals has obvious implications for the desirable optoelectrical properties relevant to highly efficient solar cells or stable materials. The use of Bi^3+^ or Sb^3+^ results in limitations in the charge transport due to the formed layered vacant structure. Ge^2+^-based halide perovskite materials exhibit poor chemical stability and poor solubility in polar solvents [17]. Cu^2+^-based halide perovskite has shown maximum efficiency of 0.99% to date due to the limitations of low absorption coefficient and high effective mass of the holes [18]. Solar cells based on Ti-halide perovskites have so far shown an efficiency of 3.3% [16]. Sn-halide perovskites (Sn-PVK) have similar or superior optical and electronic properties to Pb perovskites, such as a narrow band gap, high mobility, and long-lived hot charge carriers [19]. More importantly, Sn has low toxicity and the degradation product after exposure to air is SnO_2_. To date, Sn-PVK-based solar cells have attained around 14.81% efficiency, which is the best among the non-Pb-PVK-based solar cells [20]. Therefore, Sn halide perovskites are a promising material for developing efficient Pb-free halide perovskite solar cells.

Despite bearing such superior electronic properties, the achieved 14.81% efficiency of Sn-PVK solar cells is far inferior to the 25.7% of Pb-PVK solar cells [20]. Such a limitation arises mainly due to the different kinetics of fast crystallization and poor stability. The hindrance to the stability of Sn-PVK arises due to the facile oxidation of Sn^2+^ into Sn^4+^. This formed Sn^4+^ is responsible for unwanted high doping, leading to a high background carrier density and greatly hindering the performance of the Sn-PVK solar cells. However, Sn-PVK films with high background carrier density and high conductivity can be used as thermoelectric films. Sn-PVK films have extremely low thermal conductivity and a reasonable Seebeck coefficient [21]. Therefore, Sn-PVK films can be used as energy harvesting films, either as a photon harvesting layer in solar cells or as a waste-heat-to-energy harvesting layer in thermoelectric devices. The physical and electronic properties of Sn-PVK are discussed here from the viewpoint of benign, forward-looking alternatives to Pb-PVK. Further, we summarize improvements in the performance of 3D Sn-PVK active layers for solar cells and thermoelectric applications. Ultra-broad solar spectrum absorbers can be used to convert solar light and heat into electrical energy. They possess vast applications in information detectors and infrared thermal imaging. Such ultra-broad solar spectrum absorbance has been achieved in several ways in optimized device structures [22,23,24]. However, this is beyond the scope of this review. 

### 1.1. Structural Properties 

**Figure 1:**
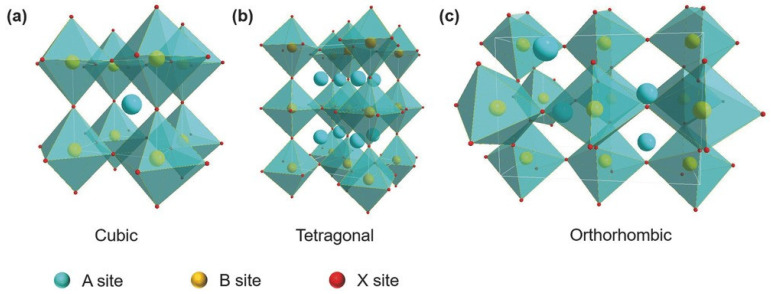
(**a**–**c**) The crystal structures of perovskite semiconductors and their symmetry. Reprinted with permission under Creative Commons Attribution License [26]. Copyright 2018, Wiley-VCH.

The feasibility of a perovskite crystal existence is decided by an empirical compound formula ABX_3_, where cation B and the halide X form a corner-sharing cubic array of BX_6_ octahedra arranged across corners. Cation A is situated within the cavities of the cuboctahedra. Therefore, the ABX_3_ perovskite crystal is formed as a cubic closed-pack array of AX_3_, with the B cation sitting at the octahedral holes. A geometric ratio called the Tolerance factor $\left(t\right)$, is often used to describe the perovskite structure feasibility and stability of the perovskite structure.

(1) $$t=\frac{r_{A}+r_{B}}{\surd 2\left(r_{B}+r_{X}\right)}\;$$

where $r_{A}$, $r_{B}$ and$\;r_{X}$ are the radii of the A-site cation, the B-site cation, and the X-site halide, respectively. Perovskite crystal structures generally exist for 0.9≤t≤1. A tolerance factor of close to 1 indicates that the perovskite crystal adopts a symmetric structure. A tolerance factor of less than 0.9 indicates an asymmetric structure and a tilting of the BX_6_ octahedra. The calculated t of the different Sn-PVK crystals are shown in Table 1. It should be noted that t varies depending on the cation of the A site. The small radius Cs^+^-based perovskite has the lowest t and the largest radius FA^+^-based perovskite has the highest t. This means that t is a measure of how perfectly the A site cation fits into the crystal lattice, and may also affect the stability of perovskite crystal. A second parameter called the octahedral factor $\left(\mu \right)$, $(\mu =\frac{r_{B}}{r_{X}}$) is defined to estimate the fit of the B cation into BX_6_ octahedra. A $\left(\mu \right)$ of between 0.442 and 0.895 estimates the possibility of obtaining the perovskite structure. The different possible perovskite structures cubic, tetragonal, and orthorhombic, resulting from the different arrangement or inclination of octahedra are shown in Figure 1.

**Figure 2:**
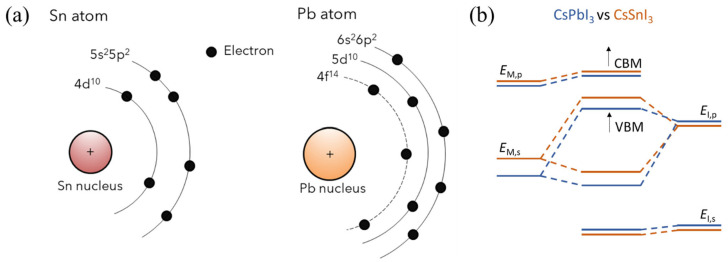
(**a**) Schematic diagram showing the lanthanide shrinkage in Sn and Pb atoms, reproduced with permission [28], Copyright 2021, American Chemical Society. (**b**) Energy level shifting upon substitution of Pb by Sn. Reproduced with permission [29], under a Creative Commons Attribution Non-Commercial License 4.0 (CC BY-NC). http://creativecommons.org/licenses/by-nc/4.0/ accessed date (accessed on 19 October 2022).

Sn-PVKs are unstable compared to Pb-PVKs because Sn has two active and easily lost 5s electrons. Compared to Pb, Sn has lower electronegativity, shallower energetics, and a smaller splitting of s and p. The Sn atoms lack lanthanide shrinkage, and the binding energy is much lower. For this reason, Sn-PVKs easily oxidize even under an inert or controlled oxygen atmosphere. In contrast, the two 6s electrons of Pb exhibit lanthanide shrinkage or an inert pair effect and are difficult to detach. Lanthanide shrinkage is defined as the high reduction in radius with enhancement in atomic number for post-lanthanide elements. Such typically pronounced behavior of the s orbital electrons alters the reactivity of Sn compared to Pb. The reaction rates of SnI_2_ with Lewis bases such as MAI and FAI are faster than those of PbI_2_. The active 5s electrons of Sn are responsible for the higher Lewis acidity of SnI_2_. Figure 2a shows the lanthanide shrinkage effect in Sn and Pb atoms. When Pb is replaced by Sn, the ionization energy decreases, and the perovskite material gains shallow energetics (Figure 2b). Shi et al. [22] performed density functional theory (DFT) calculations to deduce the changes in the electronic behaviors of MASnI_3_ and FASnI_3_. They found that the antibonding coupling between Sn 5s and I 5p in FASnI_3_ becomes weaker due to the larger size of FA. This leads to higher formation energy of Sn vacancies/defects in FASnI_3_. [25] This finding explains the stable and reproducible performance of vacancies and defects in FASnI_3_-based solar cells. 

### 1.2. Electronic and Optical Properties 

To utilize the semiconducting properties of perovskites for photo harvesting and thermoelectricity applications, one must understand the electronic properties. The electronic properties are mainly determined by the position of the valence band maximum (VBM), and the conduction band minimum (CBM), which determine the band gap energy, dielectric constant, effective mass, carrier mobility, and free charge carrier density. In this section, we first discuss these points.

According to the detailed balance limit, single junction solar cells with 1.34 eV bandgap energy can reach a photoconversion efficiency of 33.16% at AM 1.5 solar spectra. MASnI_3_, CsSnI_3_, and FASnI_3_ typically have bandgap values in the range of 1.1 to 1.4 eV, allowing absorption in the visible and infrared spectra. It is known that MASnI_3_, FASnI_3,_ and CsSnI_3_ are direct bandgap semiconductors where the maximum and minimum of the valence band coincide on the reciprocal space position. 

**Figure 3:**
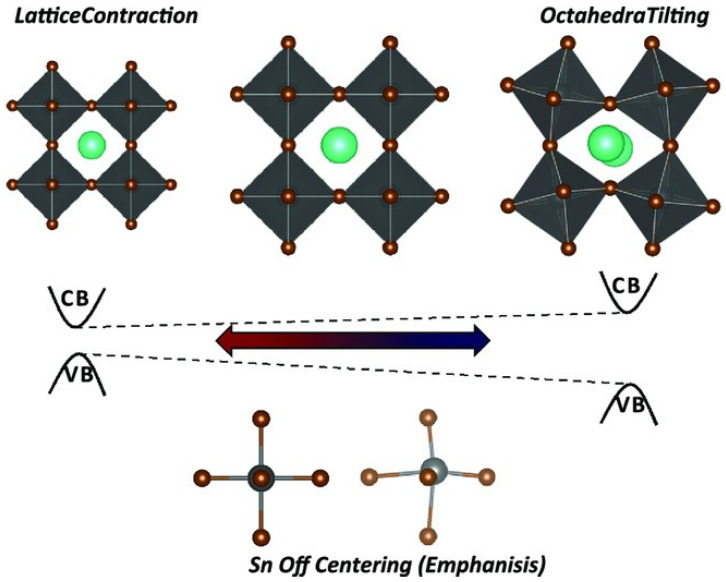
Perovskite lattice distortion. Reproduced from [31] under Creative Commons CC By license. Copyright 2018, Wiley-VCH.

**Figure 4:**
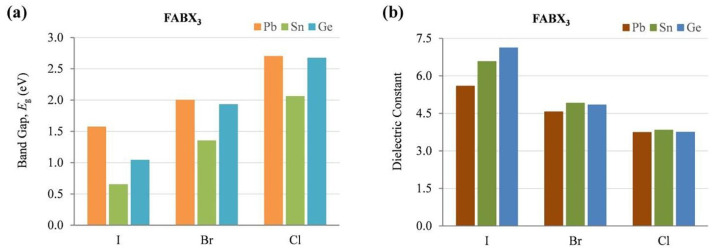
The calculated optoelectronic parameters of FABX_3_ perovskite (B = Pb, Sn, Ge; X = I, Br, Cl) (**a**) Bandgap energy, (**b**) Dielectric constant. Reproduced with permission [32]. Copyright 2019, Elsevier.

From Table 1, the optical and electronic properties should change by changing the cation of the A site when the arrangement of perovskite crystals is varied. The Sn-PVK valence band is determined by the hybridization of Sn(5s) and I(5p) antibonding orbitals, and the conduction band is formed by the Sn(5p) and I(5p) antibonding states [30]. Therefore, the arrangement of SnI_6_ octahedra of the perovskite crystal may be responsible for the variation of the optical band gap. The variation of Sn-I bond length or Sn-I angle can cause such a change. The more Sn^2+^ centered in the octahedra, and the more cubic the crystal, the smaller the crystal size. This tends to result in a smaller bandgap. Conversely, the deformation of the crystal or a decrease in symmetry can cause Sn to become decentered, which also leads to a change in bandgap energy (Figure 3). [31] However, the A site cation is located in the cavity of the cuboctahedron. Thus, the A site cation indirectly affects the bandgap as it can cause tilting of the octahedron and distortion of the unit cell. A similar situation is shown in Table 2, as the FA^+^ cation is larger than the MA^+^ cation and the Cs^+^ cation, MASnI_3_ has a tetragonal structure (P4mm space group), FASnI_3_ has an orthorhombic structure (Amm2 space group), and CsSnI_3_ has an orthorhombic structure at room temperature. CsSnI_3_ has a low tolerance factor, and black γ-CsSnI_3_ can transform into yellow CsSnI_3_ at room temperature retaining the orthorhombic structure (Pnma space group). Ostrikov et al. studied the bandgap energy and dielectric constant of FABX_3_ (B = Pb, Sn, Ge; X = I, Br, Cl) [32]. Figure 4 shows that the bandgap becomes larger when X = I was replaced with Br or Cl independent of B = Pb, Sn, or Ge. The dielectric constant of FABI_3_ was higher than that of FABBr_3_ and FABCl_3_. This indicates that I-containing perovskites are more suitable for photovoltaic applications. FAGeI_3_ had the highest dielectric constant of 7.12; whereas, FASnI_3_ had a dielectric constant of 6.57. FABBr_3_ and FABCl_3_ have low dielectric constants and should be explored for various microelectronic applications.

Light incident on the photo absorption layer of the perovskite generates excitons. Excitons are electrostatic pairs of electrons and holes bound by the Coulomb force. The perovskite photo absorption layer has high dielectric properties, and Sn-PVKs are more capable of generating Wannier–Mott excitons. At ambient temperature, the exciton energy of the halide perovskite photo-absorbing layer was determined to be a few millielectron volts (meV). This shows that the photogeneration of charge carriers can occur at room temperature energy (26 meV), as the reported upper limit of exciton energy is less than 20 meV [33]. Since the exciton energy values are lower than the thermal energy of the operating temperature of photovoltaic devices, free charge carriers and not excitons are photogenerated. The low exciston energy of Sn-PVK may be due to the exceptionally low effective mass of electrons (m_e_) and holes (m_h_) (Table 2). The charge carrier mass is an important parameter determining the optoelectronic properties of Sn-PVK. It can be calculated from the dispersion of the electronic band curvature at VBM and CBM. Lambrecht et al. calculated the band gap, exciton energy, and effective mass of CsSnX_3_ (X = I, Br, Cl) [30].

**Figure 5:**
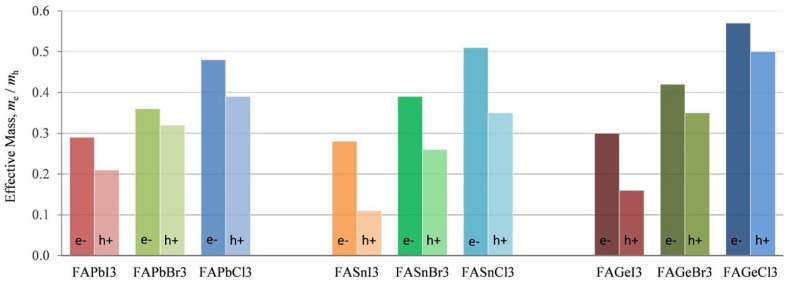
The comparison of the calculated effective mass of FABX_3_ perovskite (B = Pb, Sn, Ge; X = I, Br, Cl). Reproduced with permission [32]. Copyright 2019, Elsevier.

Table 3 shows the effective mass of CsSnX_3_. CsSnI_3_ has the lowest effective mass, which explains the reported high value of hole mobility of 585 cm^2^/V·s [35]. Substitution by X = Cl/Br increases the effective hole mass, making them unsuitable for photovoltaic applications. The low effective mass also contributes to high mobile charge carriers. Ostrikov et al. [32] investigated the effective mass of FA-based semiconductors using a DFT study (Figure 5). The effective mass of holes and electrons increased with the substitution of Br or Cl for I, regardless of the perovskites Pb, Sn, or Ge. It is also found that among the Pb-free materials FABX_3_ (B = Sn, Ge; X = I, Br, Cl), FASnI_3_ has the lowest effective mass of holes and is, therefore, suitable for photo harvesting. Ge-based perovskites have the highest effective mass, while Pb and Sn-based perovskites have almost similar effective mass.

We can conclude from these results that the degree of antibonding mixing of the p orbital of the anion in the VBM decreases from Cl to Br to I as the p levels of anions move farther down. This explains the change in bandgap energy and effective mass. In the distorted tetragonal and orthorhombic structure, the bond angle of M-X-M deviates from 180°, leading to a decrease in the orbital overlap. This tends to increase the effective mass. 

**Figure 6:**
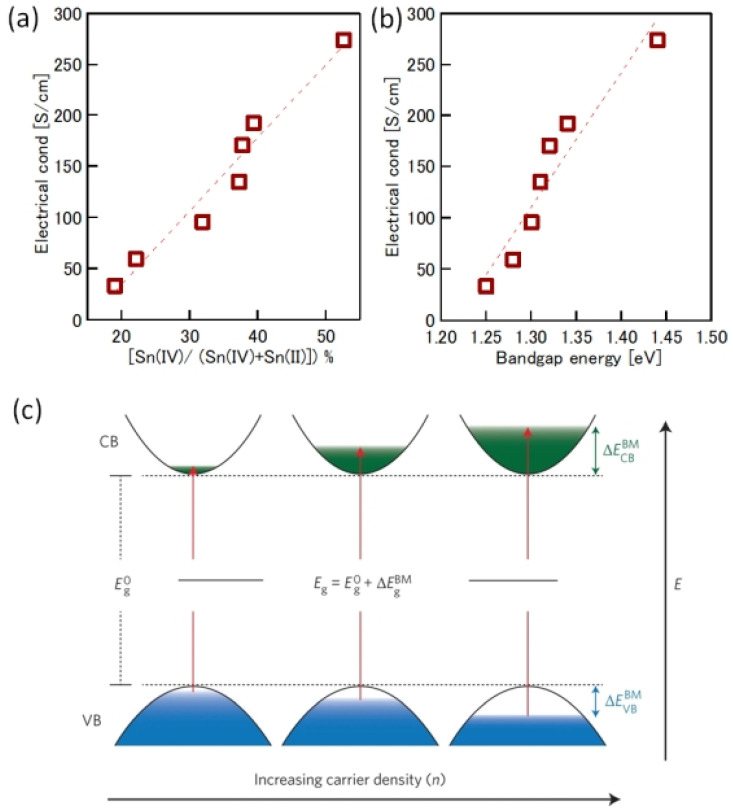
Correlation between (**a**) Sn^4+^ and electrical conductivity, (**b**) bandgap energy and electrical conductivity. Reproduced with permission [40]. Copyright 2020, American Chemical Society. (**c**) Schematic representation of the Burstein–Moss effect showing the defects, bandgap, and charge carrier density relationship. Reproduced with permission [41]. Copyright 2014, Springer Nature.

One of the obvious problems with tin halide perovskites is the facile oxidation of Sn^2 +^ /Sn^4+^ and the concomitant formation of Sn vacancies. SnF_2_ has become an essential additive to avoid such easy oxidation. Kahmann et al. [36] reported that the optical properties of SnF_2_-doped FASnI_3_ film have a better lifetime of 1.5 ns compared to the 220 ps of the pure FASnI_3_ film. The photoluminescence (PL) peak of SnF_2_-doped FASnI_3_ film was at 1.347 eV, while the pristine film had a peak at 1.351 eV. PL spectra of SnF_2_-FASnI_3_ film had a suppressed width peak of 107 meV compared to the 115 meV of the pure FASnI_3_ film. Such a shift in the peak is due to the partial filling of the valance band by the use of SnF_2_. Milot et al. [37] showed that the addition of SnF_2_ suppresses the background charge density of FASnI_3_ thin films. Pure FASnI_3_ thin films exhibited a charge density of 2.2 × 10^20^ cm^−3^. SnF_2_ doping (10%) suppressed the background charge density to 7.2 × 10^18^ cm^−3^. The absorption spectra were blue-shifted in the pure FASnI_3_ thin film. Baranwal et. al. showed that continuous stirring of Sn-PVK precursor under DMSO as co-solvent led to a blue-shift of the absorption spectral edge of films [38]. Wakamiya et al. also showed that the pure FASnI_3_ film exhibited a blue-shifted PL peak without the addition of SnF_2_ [39]. These reports show that the absorption and photoluminescence spectral peaks depend on the quality of the film. Films with Sn defects and high background carrier density show blue-shifted spectra. Recently, the electronic properties of Sn-PVK were studied in various nanocomposite structures in which Sn-PVK was coated on different metal-oxide layers (metal-oxide = Al_2_O_3_, NiO_x_, SnO_2_, TiO_2_, ZnO, and ZrO_2_). The electrical conductivity, bandgap energy, and Sn^4+^ content were greatly varied in these nanocomposite structures of CsSnI_3_/metal-oxide [40]. However, a direct correlation between electrical conductivity, band gap energy, and Sn^4+^ defects was observed (Figure 6a,b). This anomalous behavior of Sn-PVKs for changes in the Sn^4+^ defects leading to changes in the carrier density and bandgap energy has been explained by the Burstein–Moss effect, as shown in Figure 6c. 

### 1.3. Defect Physics and Charge Transport

The Sn^2+^ of the Sn-PVK materials is very susceptible to oxidation to form Sn^4+^ (Figure 2a). This undesirable oxidation leads to a vacancy-type degraded Sn-PVK structure Cs_2_SnI_6_ [42]. In this process, Sn-PVK suffers from intrinsic Sn2p vacancy because the antibonding coupling of Sn 5s and I 5p atomic orbitals is very strong. As the antibonding coupling of Sn 5s and I 5p atomic orbital is very strong, the formation energy of Sn2p vacancies is lower. This significantly deteriorates the semiconducting behavior and causes high electrical conductivity of Sn-PVK due to self-doping. However, while vacant Sn2p and formed Sn^4+^ limit the performance of solar cells, they are far-reaching for thermoelectric devices.

**Figure 7:**
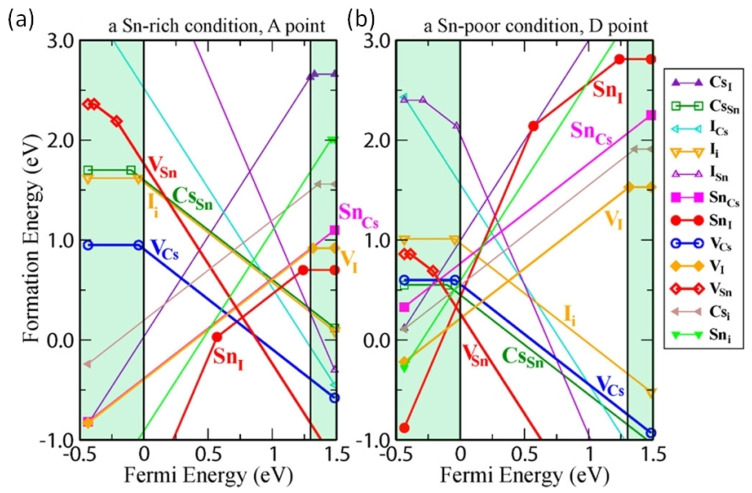
(**a**) Calculated formation energy of defects under Sn rich condition, (**b**) Calculated formation energy of defect states under Sn poor condition; The shaded region below the fermi energy shows the valence band and the shaded region above the fermi energy shows the conduction band. Reproduced with permission [43]. Copyright 2014, American Chemical Society.

**Figure 8:**
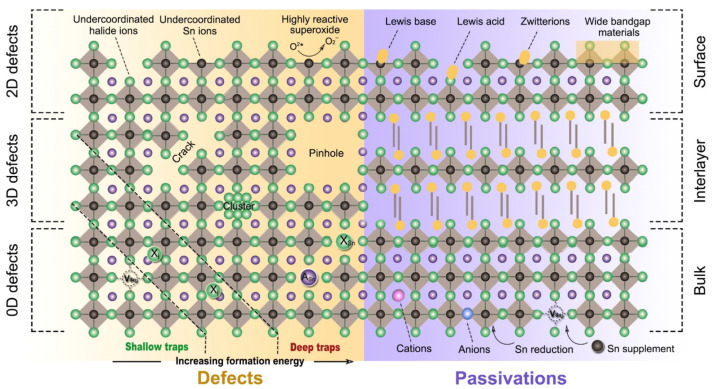
Possible defect states and passivation strategy in Sn-PVK films. Reprinted with permission [46]. Copyright 2020, American Chemical Society.

Wei et al. used a first-principles study to show that controlling the intrinsic defects of Sn-PVK is crucial (Figure 7a) [43]. Under low-Sn conditions, deep defects can form from Sn and Cs vacancies. These acceptor defects trap the charge carriers and produce high p-type conductivity and background charge density. Such a condition is ideal for the fabrication of thin films for thermoelectric applications. Conversely, under Sn-rich conditions, acceptor defects leading to high background charge density can be controlled. Therefore, the p-type defects can be controlled to use the Sn-PVK for photo-harvesting applications in solar cells. A Sn-rich Sn-PVK condition is used for solar cell applications, which minimize the background charge density employing SnF_2_ as a common additive [44]. However, further Sn-rich conditions lead to deep defect formation of $Sn_{I}$. This e-acceptor defects trap the electrons and cause nonradiative e-h recombination. Herz et al. studied the conductivity spectra of FA_0.83_Cs_0.17_SnI_3_ thin films without photoexcitation [45]. SnF_2_ was added to the precursor solution. SnF_2_ as an additive can overcome the dark conductivity by suppressing Sn^2+^ oxidation and limiting Sn vacancies. However, the dark current was still very high. Figure 7 also shows that Sn-PVK tends to form defects in a facile manner despite the Sn-rich or Sn-poor condition. Such defects are schematically shown in Figure 8. Their possible passivation strategy is also demonstrated. 

The formation of such an easy defect energy state determines the photoelectric properties of the Sn-PVK semiconductor adversely. These trap states inevitably affect photovoltaic performance and stability. 

## 2. Solar Cells

The J-V characteristic of a solar cell is determined by

(2) $$J\left(V\right)=J_{P}-J_{0}\left[exp\left(\frac{q\left(V+JR_{s}\right)}{nkT}\right)-1\right]-\frac{V+JR_{sh}}{R_{sh}}$$

where $J_{P}$ is the generated photo current density upon photo-illumination, $J_{0}$ is dark saturation current density, $R_{s}$ is the series resistance of the functional device, $R_{sh}$ is the shunt resistance of the photo active layer, n is the diode constant, and J and V denote the current density and voltage of the solar cells, respectively. The output current of the solar cell is determined from Equation (2). The generated photocurrent in the solar cells is obtained by subtracting the resistance loss (series and shunt) and diode loss.

The photoexcited carriers are trapped at the defects of the undercoordinated bonds, vacancies, and pinholes in the bulk or the surface, or at the grain boundary (Figure 8). This suppresses the collected photocurrent $J\left(V\right),$ and reduces the photovoltaic performance. In addition, $\;R_{sh}$ and $R_{s}\;$ are adversely affected, resulting in poor FF and poor Voc. Therefore, various strategies, such as additive, substitution, surface passivation, and low-dimensional 3D-2D of Sn-PVK, have been employed to suppress the defects and develop efficient Sn-PVK solar cells with high photoelectric performance. Table 4 shows the measured electronic parameters of the Sn-PVK layer in the solar cell structure. The polycrystalline MAPbI_3_ film has an intrinsic carrier density of 10^15^/cm^3^ and a diffusion length of 0.1 to 2.7 µm [19,47]. The photoactive layers of Sn-PVK have comparable charge density but shorter diffusion lengths than Pb-PVK (Table 4). This strongly affects the thickness of the active layer, as a thinner Sn-PVK of ~200 nm is generally employed in solar cells. Conversely, Pb-PVK photo absorption layers are ~500 nm thick. This thin Sn-PVK limits the photon yield and photoelectric performance. This can be related to the lower diffusion length of the Sn-PVK photo-active layer. The charge carrier diffusion length is a measure of how long charge carriers can diffuse without interruption. Acceptor defects of Sn-PVK trap the photogenerated charge carriers and thus limit the diffusion length. Snaith et al. [48] have shown that the charge carrier density and diffusion length are correlated. The Sn-PVK film with a high back ground charge density of 10^18^/cm^3^ exhibited 20 nm, while the diffusion length was 1 µm for a low charge density of 10^15^/cm^3^. This illustrates that the diffusion length and the lifetime of charge carriers depend on the quality and defects of the film. 

**Figure 9:**
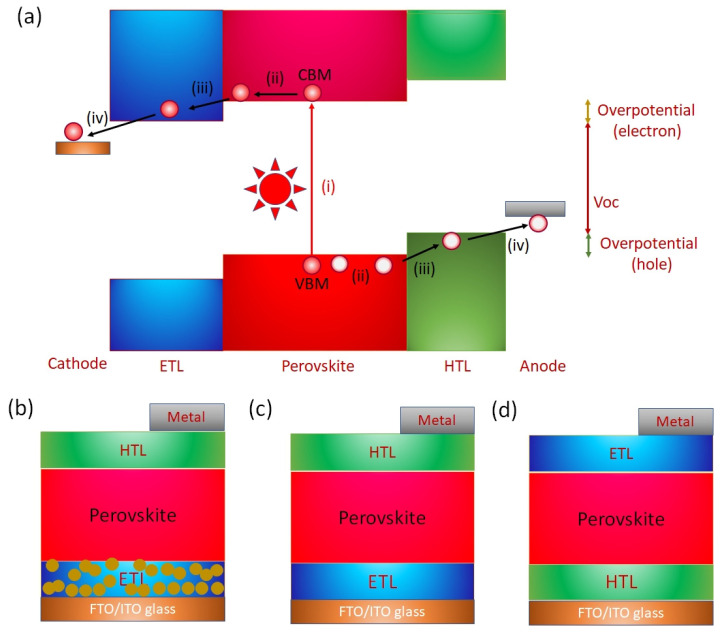
(**a**) Schematic diagram showing the working principle of perovskite solar cells, (**b**) Structure of normal/regular mesoscopic n-i-p solar cells, (**c**) Structure of normal structure planer n-i-p solar cells, (**d**) Structure of planer p-i-n (inverted) solar cells.

The operation of a PVK solar cell involves (i) Absorption of photons and generation of charge carriers; (ii) charge transport within the bulk; and (iii) charge extraction at the interfaces (Figure 9a). The photons with energy greater than the difference of the VBM and CBM of the PVK layer are incident. The photoexcited excitons are generated. Such photoexcited excitons have relatively low binding energy. The PVK layers have a high dielectric constant. The low exciton energy contributes to the fact that electrons and holes dissociate immediately after exciton generation at room temperature. These dissociated electrons and holes move toward the cathode and anode under the influence of the generated built-in potential of the film. However, the defect states of the active layer lead to nonradiative recombination and badly affect the short-circuit current $\left(J_{sc}\right)$ and open circuit voltage ($V_{oc}$). In addition, recombination at the interfaces is a possible obstacle to achieving high photoelectric performance. An electron transport layer (ETL) and a hole transport layer (HTL) are used to assist in charge collection. Such charge transport layers help in charge removal and maintain the built-in potential. An ETL with a work function shallower than the Fermi level of the active PVK layer forms a downward band bending interface, and an HTL with a sufficiently deep work function is required. Such an energy level cascade/alignment between ETL, HTL, and the photoactive PVK layer is required for efficient charge transfer and the desired high Voc. Tshis alignment of energy levels enables efficient charge transfer and a high PCE. Sn-PVK surfaces have unsaturated and undercoordinated bonds, a possible center of recombination that adversely impacts the photoelectric performance. Charge recombination at the interfaces of the Sn-PVK active layer has been suppressed by various thin passivation layers at the HTL and ETL interfaces. The damage caused by the vacuum deposition of metals (Ag/Au) may be controlled by a thin bathocuproine layer. The functional diagram of a PVK solar cell is shown in Figure 9a. 

The architecture of the perovskite solar cell is shown in Figure 9b–d. Perovskite solar cells with a mesoscopic normal structure are derived from dye-sensitized solar cells (DSSC) in which nanoporous charge transport layers are first deposited on fluorine-doped transparent conductive oxide glass (FTO glass) or Sn-doped indium oxide glass (ITO glass). Generally, TiO_2_ or SnO_2_ are deposited as the ETL layer. The dye of DSSC was replaced by a photoactive perovskite layer and deposited using a solution approach or vacuum deposition. This photoactive layer forms a solid layer following the annealing process. Next, a hole-selective layer is deposited instead of the liquid electrolyte. Finally, a charge-extractive metal layer is deposited to complete the solar cell fabrication. Therefore, perovskite solar cells are also called all-solid DSSCs. The discovery of the charge transport mechanism, the long diffusion length, and the desire to simplify the solar cell structure led to the proposal of a planar structure. In solar cells with a planar structure, a photoactive perovskite layer is inserted as a thin layer between the ETL and HTL (Figure 9c,d). 

The photoelectric performance of solar cells can be improved by enhancing the Jsc, fill factor (FF), and Voc.

(3) $$Efficiency\left[\%\right]=\frac{J_{sc\;}\times V_{oc}\times FF}{P_{in}}\times 100\;$$

Among the photovoltaic parameters, FF is decided by the perfectness of the squareness of the J-V curve, and $V_{oc}$ is the maximum open circuit voltage. These photovoltaic parameters are affected by the defect states and nonradiative recombination loss center to induce passive losses in $R_{s}$ and $R_{sh}$ and recombination loss at the interfaces. 

**Figure 10:**
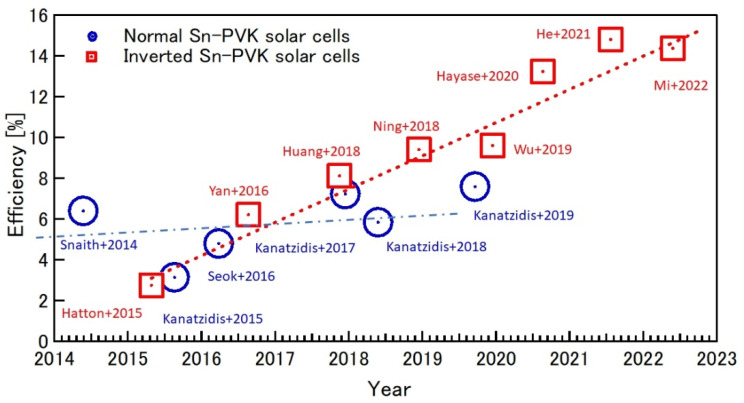
Efficiency evolution of Sn-PVK solar cells in regular and inverted structures over the years. Inverted structure solar cells have reached a maximum of 14.83%, while the normal structure has, to date, reached 7.59%. (Snaith+2014 [48]; Hatton+2015 [61]; Kanatzidis+2015 [62]; Seok+2016 [63]; Yan+2016 [64]; Kanatzidis+2018 [65]; Kanatzidis+2017 [66]; Huang+2018 [67]; Kanatzidis+2019 [68]; Ning+2018 [69]; Wu+2019 [70]; Hayase+2020 [53]; He+2021 [20]; Mi+2022 [51]).

The Summary of the efficiency evolution in recent years of Sn-PVK in normal and inverted structures is shown in Figure 10. The Sn-PVK solar cells with inverted structure, first introduced in 2015, shows continuous progress in improving efficiency, while the photoelectric performance of Sn-PVK solar cells with normal structure has not witnessed similar growth. 

### 2.1. Normal Structure

**Figure 11:**
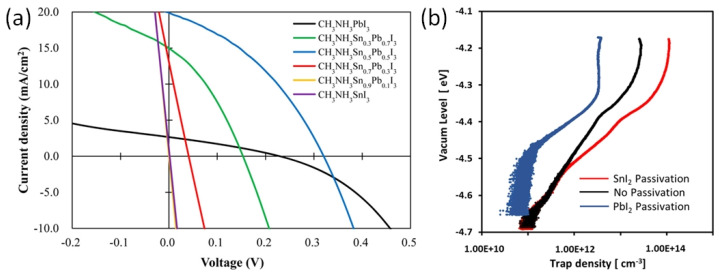
(**a**) Short–circuited J–V characteristics of MASnI_3_ photoactive layer-based normal structure solar cell (purple color), reprinted with permission [71]. Copyright 2014, American Chemical Society. (**b**) Trap density evaluation of TiO_2_ layer passivated by SnI_2_ and PbI_2_. reprinted with permission [73]. Copyright 2020, American Chemical Society.

In solar cells with normal structure, the perovskite ink is deposited on electron-selective metal oxide layers consisting of TiO_2_ or SnO_2_. It is worth noting that these metal-oxide layers are porous with a common phenomenon of oxygen vacancies. These vacancies may serve as a trap center to adversely affect the photoelectric performance. Ogomi et al. showed that the MASnI_3_ semiconducting layer has no photoelectric properties in solar cells with an n-i-p structure and TiO_2_ electrodes (Figure 11a) [71]. The first report on Sn-PVK solar cells was published by Snaith et al. in 2014. The photo-harvesting layer of MASnI_3_ was used to fabricate the cells in an n-i-p structure on TiO_2_ electrodes. A maximum efficiency of 6.4% at 0.88 V $V_{oc}$ was achieved. However, looking closely at the distribution bar, the average efficiency was less than 2% and most of the solar cells had an efficiency close to 0% [48]. Zhao et al. reported solar cells based on MASnI_3_ and FASnI_3_ in a normal n-i-p structure. The cells were fabricated as FTO glass/cp-TiO_2_/mp-TiO_2_/Sn-PVK/spiro-MeOTAD/Au. However, poor diode rectification performance was obtained with efficiencies of 0.16% and 0.65%, respectively [72]. 

The reason for this unusual behavior of poor diode characteristics or short-circuit in Sn-PVK solar cells with normal structure was investigated by Hamada et al. [73]. Using, a thermally stimulated current, they showed that when the inorganic TiO_2_ layer was passivated by Sn-PVK, the defect states were largely enhanced (Figure 11b). In contrast, TiO_2_ passivated with Pb-PVK showed suppressed defect states. This poor photoelectric performance of Sn-PVK in n-i-p-based solar cells due to the enhanced defect density with metal oxide films led to this structure attracting less interest from researchers. This can be evident from Figure 10 as the normal structure Sn-PVK solar cell’s efficiency evolution rate is slower than that of inverted Sn-PVK solar cells. 

**Figure 12:**
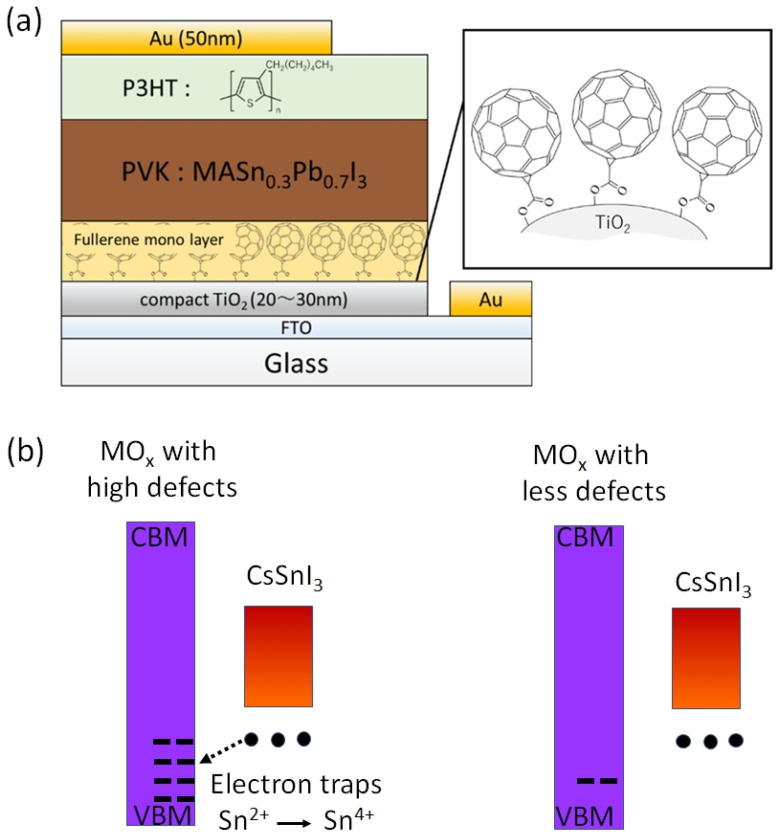
(**a**) The interlayer of C60-COOH was used to avoid the effect of acceptor defects of the TiO_2_ layer. Reprinted with permission from [73]. Copyright 2020, American Chemical Society. (**b**) Mechanism of oxidation of Sn-PVK resulting in short-circuiting or poor rectification behavior, in normal structure solar cells. MOx represents metal oxide. Reprinted with permission from [40]. Copyright 2022, American Chemical Society.

Jen et al. fabricated Sn-PVK solar cells with normal and inverted structures [74]. They showed that the addition of trimethyl iodide in FASnI_3_ succeeded in achieving a high efficiency of 7.09% in an inverted structure and only 4.43% efficiency was obtained in a normal structure. However, the point is that a thin C60-coated electron-selective layer SnO_2_ layer was used in the normal-structure solar cells. With this modified electron transport layer, they succeeded in fabricating functional Sn–PVK solar cells. Xu et al. developed {en} FASnI_3_-based solar cells in normal n-i-p structure [75]. They used an inter-layer of C60-pyrrolidine over inorganic SnO_2_. The double-layer SnO_2_-C60 pyrrolidine tris-acid was used as an electron transport layer. The performance of the solar cell was improved to 7.40% with a Voc of 0.72 V and a Jsc of 16.45 cm^2^/V·s. However, the solar cells fabricated on bare SnO_2_ ETL showed poor performance with a Voc of ~0.2V and Jsc of ~2 mA/cm^2^. Such a C60-pyrrolidine over inorganic SnO_2_ was able to suppress the charge recombination. Hamada et al. showed that the use of C60-COOH as an interlayer between the photoactive Sn-Pb PVK layer and the TiO_2_ electrode is essential for functional n–i–p-based Sn-Pb solar cells (Figure 12a) [73]. This C60-COOH interlayer strategy resulted in an enhanced photoelectric performance of 7.91% compared to 5.14% of bare SnO_2_ electrodes. They concluded that without the C60-COOH interlayer, Sn-Pb PVK was itself oxidized to form appreciable amounts of Sn^4+^. The reason for this Sn^4+^ formation with metal oxide and Sn–PVK contact was recently investigated by Baranwal et al. [40,76].

Baranwal et al. reported that Sn-PVK coated on an inorganic Y_2_O_3_ layer exhibited enhanced electrical conductivity due to the formation of Sn^4+^ by the oxidation of Sn^2+^ [76]. This was also confirmed by the lowering of the Seebeck coefficient values. This behavior is similar to that of the normal Sn-PVK solar cells with an n-i-p structure, where poor rectification or short-circuiting was observed due to Sn^4+^ formation without an interlayer at the metal oxide/PVK interface. This unusual Sn-PVK oxidation on the inorganic metal oxide layer was explained by an ultraviolet photo spectroscopy study that showed that the inorganic metal-oxide layers have sufficient defect energy states in the bandgap, above the VBM, and below the work function energy level. These electron acceptor defect states easily attract and trap the electrons from shallow energetic Sn-PVKs. Sn-PVKs tend to lose electrons easily, and, therefore, Sn-PVK becomes positively charged and Sn^4+^ is formed immediately even if the fabrication process is done in an inert or N_2_ atmosphere [40]. It was concluded that the reason for the poor efficiency and limited reports of n-i-p-based Sn-PVK solar cells with normal structure was the oxidation of Sn-PVK due to the defect states of the metal oxide layers (Figure 12b). In the laboratory, we can fabricate FASnI_3_ solar cells with an inverted structure and PEDOT:PSS as the HTL with an efficiency higher than 10% [53,56]. However, Sn-PVKs with Al_2_O_3_, NiO_x_, TiO_2_, SnO_2_, ZrO_2_, and ZnO electrodes always show high conductivity leading to poor solar cell performance [40,76]. Padture et al. have reported a similar result [77]. They showed that, when GeI_2_ was added in the FASnI_3_, a compact layer of GeO_2_ was established at the interface of NiO_x_/FASn_0.9_Ge_0.1_I_3_. Such a compact GeO_2_ was critical for suppressing Sn vacancies and Sn^2+^ oxidation, as it avoided the direct contact of Sn-PVK and NiO_x_. Miyamoto et al. investigated the effects of the nanoporous electron transport layer on the performance of Sn-PVK solar cells in an n-i-p structure [78]. They controlled the pore size of the Nb_2_O_5_ ETL and concluded that the decreased pore size of the ETL resulted in the faster collection of photogenerated charge carriers, which led to an improved photoelectric performance. However, a detailed study of the physical interaction of used 2D-3D Sn-PVK (FAPEASnI_3_) and the pore size of Nb_2_O_5_ is needed. A FAPEASnI_3_-based photoactive layer deposited on Nb_2_O_5_ as an ETL with a pore size of 5 nm resulted in 7% efficiency. 

### 2.2. Inverted Structure

To date, Sn-PVK solar cells with high efficiency have been described in inverted structure with PEDOT:PSS as the HTL. SnF_2_ is the most common component of the precursor to compensate for the Sn defects. Zillner et al. found an interesting result [79]; they investigated the effect of SnF_2_ addition on FASnI_3_ perovskite solar cells. It was found that SnF_2_ accumulates at the top of PEDOT:PSS. Sn of SnF_2_ and S of PEDOT:PSS preferentially form a 1.2 nm thin interlayer of SnS at the interface of PEDOT:PSS/FASnI_3_. However, this intermittent layer of SnS was not formed when the mixed Pb-Sn perovskite FA_0.75_MA_0.25_Sn_0.50_Pb_0.50_I_3_ was used. 

#### 2.2.1. Device Engineering 

**Figure 13:**
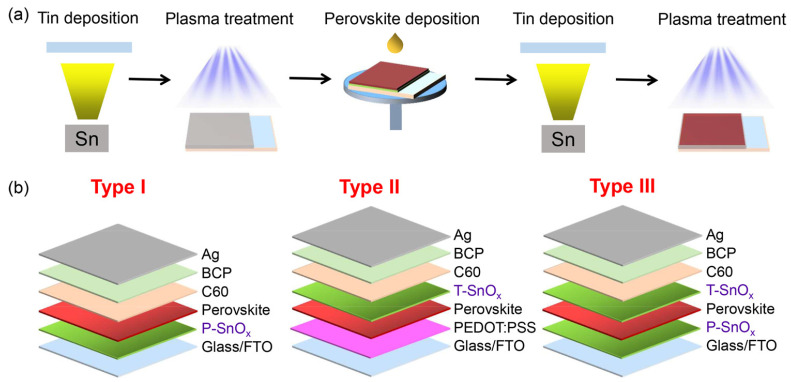
(**a**) The Sn-PVK solar cells fabrication process using plasma-treated tin as a SnOx film working as an ambipolar layer. (**b**) Role of plasma-grown SnOx was studied in three different solar cell structures, either as a HTM or perovskite protective layer or the combination of both. Reproduced with permission [49]. Copyright 2022, American Chemical Society.

Wang et al. investigated a new device structure using inorganic SnO_x_ as an ambipolar layer in Sn-PVK solar cells [49]. The SnO_x_ was used in situ as the bottom surface and on the top surface of Sn-PVK. Thus, SnO_x_ served two different purposes as HTL and PVK protective layers. The bottom SnO_x_ can efficiently transfer the charge carriers, and the upper SnO_x_ served as a protective layer reducing Sn^4+^ to Sn^2+^ and supporting facile electron transfer but blocking hole transfer. The SnO_x_ layer was deposited in a two-step simple process. First, Sn powder was vapor-deposited in a thermal evaporation method. Sn powder has a moderately low melting temperature of 231.9 °C. Second, this thermally evaporated tin was exposed to plasma under ambient conditions to convert to SnO_x_. The charge-conducting properties of SnO_x_ were adjusted by controlling the plasma exposure on the evaporated Sn film. This plasma exposure time adjustment varied the ratio of Sn and O of SnO_x_ to work as an ambipolar layer. The bottom and top layer SnO_x_ thicknesses were maintained at 10 and 2 nm, with plasma exposure times of 5 min and 2 s, respectively. This simple in-situ SnO_x_ strategy resulted in an efficiency of 14.09% for Sn-PVK solar cells. The device fabrication engineering process is shown in Figure 13. This report suggests that plasma-assisted metal deposition for the fabrication of various metal oxide layers should be explored for an effective charge transport layer. 

#### 2.2.2. A 2D/3D Photoactive Layer 

**Figure 14:**
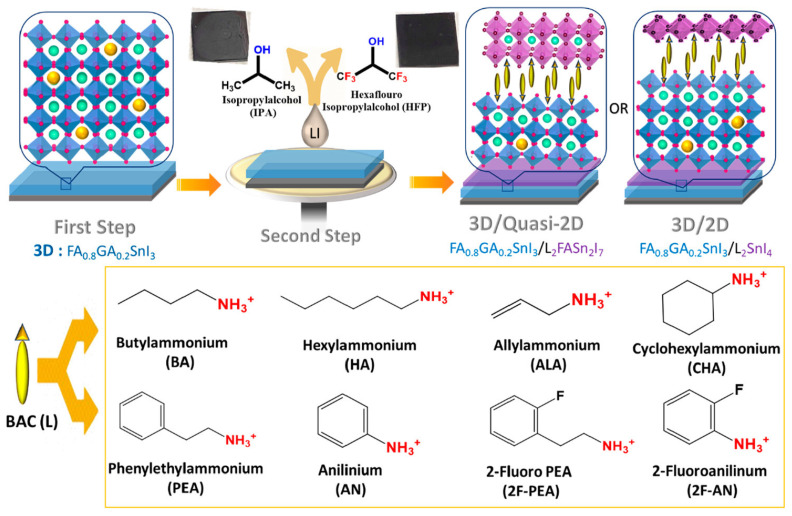
The process showing the fabrication of a 3D/quasi-2D Sn-PVK layer. Reproduced with permission under CC BY-NC-ND 4.0 [80]. Copyright 2021, American Chemical Society.

**Figure 15:**
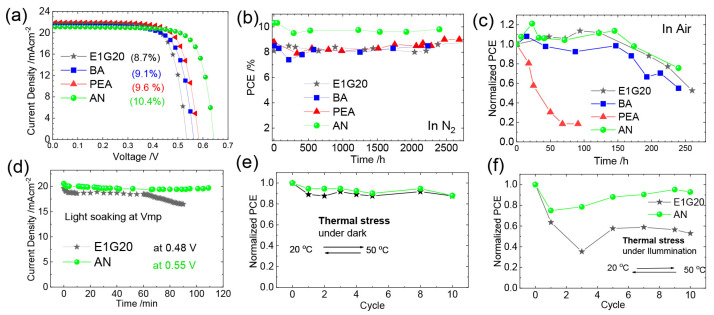
(**a**)The 3D/quasi-2D Sn-PVK layer J-V curve, (**b**) Stability under N_2_ ambient, (**c**) Air stability (**d**) MPPT test, (**e**) Thermal stress test, (**f**) Thermal stress test under illumination. Reproduced with permission under CC BY-NC-ND 4.0 [80]. Copyright 2021, American Chemical Society.

Yu et. al. introduced 4-fluoro-phenethylammonium bromide (FPEABr) as an A-site substitution in FASnI_3_ [20]. The FPEABr substitution resulted in a 2D/3D perovskite layer where, FPEABr was sitting simultaneously at the grain boundary, surface, and throughout the bulk. Thus, the bulk and interfacial defect density were suppressed. This leads to a lifetime twice that of the original FASnI_3_ film. The crystal growth was highly oriented and grain boundaries were fused with optimized 10% FPEABr. The resulting energetics showed a better energy matching, as 2D/3D perovskite had shallow VBM and CBM, approaching better alignment with PEDOT:PSS and ICBA. Due to the increased crystallinity, suppressed Sn^4+^ defects, and better energy alignment, a record efficiency of 14.81% efficiency was achieved, which is the best-reported efficiency in Sn-PVK to date. Diau et al. used a sequential deposition method to deposit various bulky ammonium cations on top of the 3D layer to form a 3D/quasi-2D layer (Figure 14) [80]. Firstly, the Sn-PVK layer was fabricated. Next, various 2D layers of butyl ammonium, hexylammonium, allylammonium, cyclohexylammonium, phenylethylammonium, anilinium, 2-fluoro phenylethylammonium, and 2-fluroannilium were coated over the PVK layer. Isopropanol (IPA) solvent was used to dissolve these 2D molecules. However, IPA damaged the Sn-PVK surface. Therefore, hexafluoro-2- propanol (HFP) was used as the solvent to avoid the dissolution of the Sn-PVK. Fluorine of HFP interacts with these 2D molecules to slow down the reactivity with the Sn-PVK surface, thus forming a 2D or quasi-2D layer. Therefore, an ultrathin layer of 2D was capped on the Sn-PVK surface covering the grain surface and grain boundary. The anilinium-based Sn-PVK bilayer photoactive device exhibited an enhanced efficiency of 10.6%. The cells were tested under a broad range of possible conditions of dark storage under N_2_ and ambient, light soaking test at the maximum power point (N_2_), and ambient thermal stress test under dark and illumination (Figure 15). Excellent stability was obtained under ambient thermal stress tests (50 °C to 55 °C (10 cycles)) and cooling at 20 °C illumination tests for anilinium-based devices. The solar cells were encapsulated for these severe tests (Figure 15e,f). This was the first report to discuss thermal stress tests of Sn-PVK, which were successful due to the self-healing phenomenon of the capping layer of anilinium. It was also reported that the temperature of the solar cells following 1-h illumination exposure led to the oxidation of the silver electrodes.

**Figure 16:**
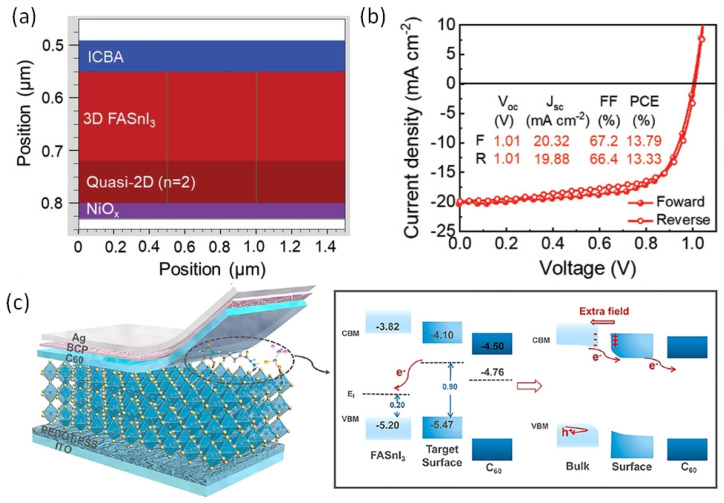
(**a**) The schematic diagram of fabricated 2D/3D Sn-PVK shows that the more soluble 2D phase was settled at the bottom of perovskite/top of NiO_x_ under the effect of vacuum, (**b**) Obtained best J-V characteristics show 1.01 V Voc irrespective of scan direction. Reproduced from [55] under Creative Commons CC BY 4.0. (**c**) Passivation of the Sn-PVK surface with 6-maleimidohexanehydrazide trifluoroacetate leads to better banding and charge extraction at C60/Sn-PVK interface. The properties of the Sn-PVK surface were changed to n-type. Reproduced with permission [83]. Copyright, 2022, American Chemical Society.

Figure 14 also shows that the vertical alignment of 2D supports facile charge conduction. To achieve vertical alignment of the triple cation-based photoactive Sn-PVK layer, the FABr incorporation strategy was followed. The 2D/3D Sn-PVK PEA_0.15_EA_0.15_FA_0.70_SnI_0.70_Br_0.30_ showed enhanced and better crystallization in the vertical plane (100), resulting in favorable energetics and leading to smooth charge conduction and improved efficiency. Following FABr substitution, the perovskite lattice shrank, and the band gap energy was improved to 1.48 eV. DFT analysis showed that the formation energy of O_2_ interstitials atoms and H_2_O were enhanced [81]. Yan et al. used a vacuum-assisted technique to fabricate stacked quasi-2D/3D PEAI-FASnI_3_ solar cells [55]. After spin coating, the films were stored under a vacuum so that solvent could be quickly removed. The more soluble 2D phase was established at the bottom, and the less soluble 3D phase solidifies at the top, forming a 2D/3D layering. The formation of the 2D capping layer at NiO_x_ was realized using simulation and is shown in Figure 16a. The 2D layer at NiO_x_ passivates the defects of NiO_x_ and supports easy hole transfer with reduced recombination loss. In addition, guanidinium thiocyanate was used as an additive, which improved the mobility and crystallinity of the 2D phase and suppressed the bulk trap density further. In this way, a record of 1.01 V of Voc was obtained regardless of forward or reverse scanning (Figure 16b) and Voc loss was minimized to only 0.39 V. An efficiency of 13.79% was reported. This reported Voc is the highest among published Sn-PVK solar cells. The cells were able to maintain 90% of their initial efficiency even after 1200 h of storage in a N_2_-filled glove box. Allylammonium cations were incorporated into the FASnI_3_ crystal to form a quasi-2D Sn-PVK. Due to the improved crystallinity in the (h00) plane, defect states were suppressed and an improved performance of 9.48% was obtained [82]. 

#### 2.2.3. Surface Passivation of Photoactive Layer

**Figure 17:**
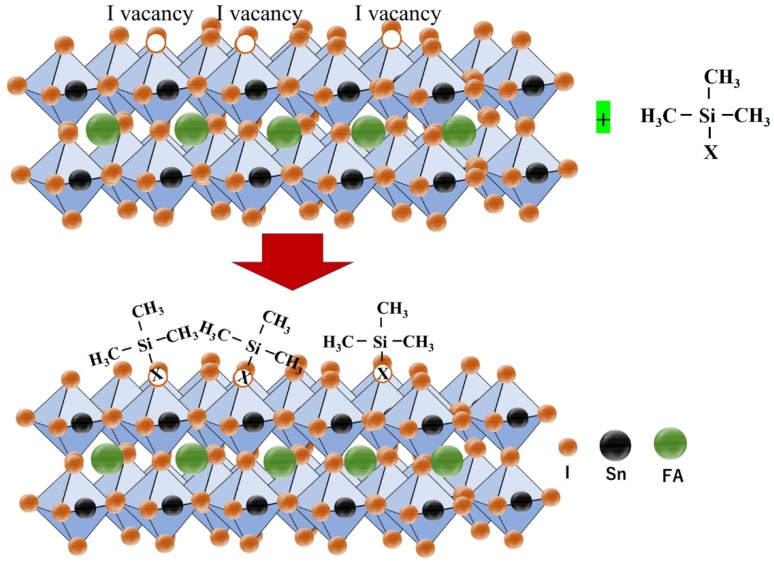
Halide passivation strategy using silyl halide as a surface passivating agent. Reproduced with permission [91]. Copyright 2022, Elsevier.

Akmal et al. used the Lewis base ethane-1,-2diamine (edamine) as a post-treatment agent to passivate the surface defects of Sn-PVK [84]. Sn-PVK surfaces have dangling and under-coordinated bonds, and high electronegative amine of edamine was coordinated with these unsaturated bonds to control the Sn^2+^ oxidation and Sn vacancies. An optimum 0.05mM edamine passivation converts unreacted SnI_2_ into a perovskite phase and improved the surface morphology. Edamine donates electrons to reconstruct the Sn-PVK surface properties from p- to n-type. The better energetics and easy charge transport assisted in an improved efficiency of 9.37%. This post-treatment strategy of Sn–PVK using edamine was successfully replicated by various authors [53,85,86,87]. The use of edamine as post-treatment was explored in Sn-Pb perovskite solar cells also. It was revealed that the p-type properties of Sn-Pb perovskite were transformed into n-type and the built-in potential was enhanced from 0.56 V to 0.76 V. This contributed to an enhancement in Voc of 100 mV [88]. Recently, a similar post-treatment strategy was followed using 6-maleimidohexanehydrazide trifluoroacetate as a surface cationic and anionic passivator [83]. The reductive nature of the hydrazine and carboxyl group neutralize the charged defects states and changed the properties of the Sn-PVK surface from p to n-type. The n-type surface leads to a better band-bending between the surface and bulk of Sn-PVK, assisting better electron transfer at the C60/FASnI_3_ interface and prohibiting hole recombination (Figure 16c). This strategy resulted in a 13.64% efficiency in FASnI_3_-based solar cells. Lia et al. used a low-dimensional phenylammonium bromide (PEABr) coating (dissolved in IPA) on the FASnI_3_ surface. Such thin PEABr-coated Sn-PVK films achieved improved crystallization, better energetic alignment, reduced defect density, and suppressed Sn^2+^ oxidation. A 7.86% efficiency was reported, which retained 80% of its original values during a 350 h light test under ambient conditions [89]. Liu et al. performed pretreatment of FASnI_3_ with n-propyl ammonium iodide (PAI) during the spin coating step after dripping the antisolvent and before annealing. The Sn-PVK crystallization process is very fast. To slow it down, DMSO is used as a co-solvent as it makes the SnI_2_-3DMSO complex. In this work, the solvent for PAI was a mixture of DMSO and chloroform. Therefore, the intermediate phase FASnI_3_.3DMSO was reconstituted with a better morphology. Intermediate film morphology had bright grain boundaries, which were grown in a template such as in the (100) plane. The PA cation did not form a 2D layer over the surface but was located at the grain boundary instead of the lattice. Overall, a highly crystallized FASnI_3_ film with a reduced trap density photoactive layer resulted in an efficiency of over 11% [90]. Zhang et al. performed the surface passivation of FASnI_3_ with trimethylsilyl halide (TMS-X) (Figure 17). The halide vacancies on the perovskite were passivated by the halide of TMS-X, as X was changed to Br, Cl, and I. TMS-X has ionic characteristics with TMS^+^ and X^−^ as ionic moieties, and they passivated anion and cation trap states. The VBM of TMS-X passivated Sn-PVK showed deeper VBM in the order of (untreated < TMS-Cl < TMS-Br < TMS-I). The fermi level was also suppressed and became shallower, such that (untreated > TMS-Cl > TMS-Br > TMS-I). It was postulated that the X^−^ ions passivated the I^−^ vacancies and TMS^+^ remains on the surface, such that TMS-X formed a hydrophobic protective surface over Sn-PVK. This strategy resulted in enhanced photoelectric performances of 12.22% (TMS-Br passivation), 11.68% (TMS-Cl passivation), and 11.58% (TMS-I passivation) [91]. 

Surface passivation of perovskites using a liquid solvent is a very common phenomenon. However, it has the interesting disadvantage that the film thickness decreases because the passivating solvents can dissolve the perovskite to some extent. Zhang et al. reported that the liquid passivation strategy reduces the thickness of the Sn-PVK active layer, thus affecting the Jsc values [85]. However, the use of vapor-assisted passivation can avoid such a problem. With such a strategy, better efficiency was achieved with edamine vapor passivation compared with liquid passivation. An improved diode factor of 1.43 with vapor edamine treatment was obtained compared to 1.57 for a liquid edamine-treated film. In the vapor passivation, a high Jsc of 24.05 mA/cm^2^ was revealed; conversely, the liquid-treated film showed a suppressed Jsc of 22.38 mA/cm^2^. This method showed an improved efficiency of 11.29%. A sequential strategy to passivate the defects and improve the crystallinity was explored recently using acetylacetone (ACAC) and edamine as post-treatment agents [56]. First, Sn-PVK films were prepared. In the post-treatment, these films were passivated with ACAC molecules. ACAC treatment fuses the grain boundaries and enhances the grain size under the Ostwald ripening effect. The films were placed on a hot plate to remove the solvents. In the next step, edamine vapor passivation was performed. Such edamine passivation suppressed the Sn^4+^ defects by coordinating unsaturated bonds. This dual passivation strategy resulted in a promising 13% efficiency with 0.79 V Voc. Chowdhury et al. reported large area (1.02 cm^2^) Sn-PVK solar cells with an efficiency of 6.33% [92]. After the deposition of FASnI_3_, the perovskite surface was treated with methylammonium chloride (MACl) vapor. To generate the MACl vapor, MACl powder was placed on a hot plate. This strategy resulted in homogenous and crystal defect-free FASnI_3_ film.

#### 2.2.4. A-Site Substitution

**Figure 18:**
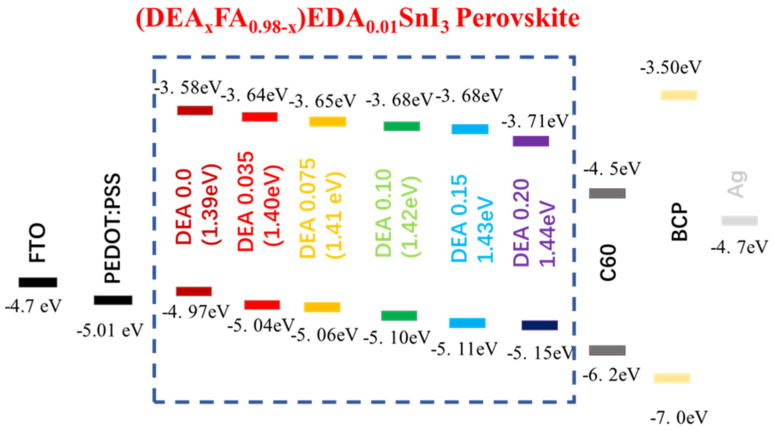
A–site substitution by DEA promotes a better energy cascade leading to improved photoelectric performance. Reproduced with permission [86]. Copyright 2021, Wiley VCH.

Bulky organic cations butylammonium iodide (BAI) and ethylene diammonium iodide (EDAI_2_) were first used as an additive by Diau et al [93]. Two-dimensional BAI improved the connectivity of grains and altered the orientation of the growth of the crystal in a preferential direction. However, fast crystallization was not suppressed, and a large number of pin holes were visible on the surface. To sustain the defects and vacancies of Sn-PVK and to slow down the crystallization rate, EDAI_2_ as an additive was proposed. In addition to this, EDAI_2_ served the purposes of retaining smooth surfaces by controlling nucleation and growth and suppressing the surface defects, and improving stability, as EDA^2+^ may occupy the vacant position of two FA^+^ ions. However, various efficient Sn-PVK solar cells are reported with the substitution of FA^+^ by EDA^2+^ [53,56,94]. Chen et al. used EDABr_2_ as a substitute at the A-site in the fabrication of FASnI_2_Br-based wide-bandgap solar cells [95]. This results in smooth and low-defect perovskite film with enhanced efficiency of 4.48%. Diau et al. replaced FA with azetidinium (AZ) [96]. Both cations have a similar radius of 253 pm and 250 pm, however, AZ has a high dipole moment. The crystallization speed of Sn-PVK was quick because of the strong interaction of AZ and SnI_6_ octahedra. Therefore, a new antisolvent trifluorotoluene (TFT) with a high dipole moment (D) of 2.86 was used in place of traditional chlorobenzene antisolvent (1.69 D). TFT produced uniform nucleation seeds and provided sufficient nucleation growth. This strategy resulted in a uniform and crystalline Sn-PVK surface. A better photovoltaic performance of 9.6% was obtained due to the increased band gap energy, band alignment, and improved crystal growth. The crystallized Sn-PVK had a much smoother surface and lower defect density resulting in better charge transport. In this report, it was shown for the first time that the shelf life of Sn-PVKs could maintain 90% of their original efficiency at 55% humidity for 15 days. Nishimura et al. used an ethyl ammonium cation to replace the A-site FA [53]. The resulting film exhibited an aligned energy cascade as the VBM and CBM become deeper, and achieved an efficiency of over 13% due to suppressed defect states. They also show that the Sn-PVK has halogen defects on the surface. Zhang et al. used diethyl ammonium iodide (DEAI) to replace the A cation [86]. The DEA-substituted Sn-PVK films exhibited larger grain sizes and deeper energetics. Higher efficiency was observed due to the suppression of Sn^4+^ and better energy alignment (Figure 18). Chen et al. optimized the A-site of FASnI_2_Br by replacing FA with guanidinium bromide [97]. Such an A-site substitution promotes a pin-hole-free surface, better energy alignment, and charge extraction at the interfaces. An improved efficiency of 7% at 0.64 Voc was reported for the wide band gap FASnI_2_Br PVK solar cell. 

#### 2.2.5. Vacuum-Assisted Strategy

**Figure 19:**
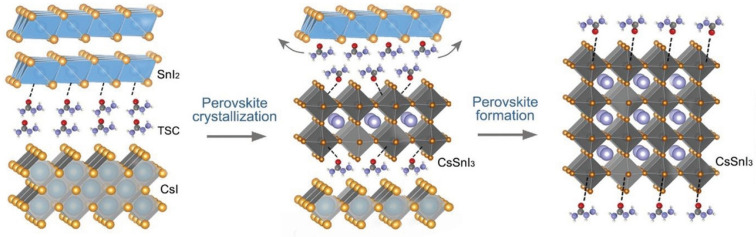
Two-step fabrication strategy of CsSnI_3_ using SnI_2_ and CsI vapor deposition with a thiosemicarbazide as a passivation layer. Reproduced with permission [99]. Copyright 2021 Wiley-VCH GmbH.

Zhao et al. fabricated Sn-PVK using an antisolvent-assisted strategy [98]. After spin coating, the films were not annealed in this work. In place of annealing, the authors stored the films under a vacuum for different time intervals. It was determined that the boiling point of Dimethylformamide (DMF), Dimethylsulfoxide (DMSO), and chlorobenzene were much higher than the film baking temperature of 70 °C. Therefore, these solvents must remain as a residue in the film. The authors show that, after prolonged storage in a vacuum, these solvent residues were evaporated completely. By using the strategy of storing the films for 12 h under a vacuum, before depositing C60 and BCP, a solar cell with a performance of over 10% was fabricated. Such a strategy was able to suppress the defect states of Sn-PVK. However, such a long-time vacuum treatment strategy to evaporate solvents is not practical despite the promise of suppressed bulk and surface defect densities. Yin et al. fabricated CsSnI_3_-based solar cells using a thermal vapor passivation strategy [99]. First, SnI_2_ was thermally deposited on a PEDOT:PSS film. Then, various passivation layers of thiourea, thioacetamide, thiosemicarbazide (TSC), and guanidine hydroiodide were deposited by a thermal evaporation method. Finally, CsI was thermally deposited and annealing was carried out. Among the passivation layers, TSC was anchored as a Lewis acid-base reaction between the S=C-N functional group and Sn ion. This strong coordination anchoring leads to an electron cloud on defects and improved the defect formation energy. During the annealing process, the perovskite precursor overcame the energy barrier, and CsI and SnI_2_ reacted to form the CsSnI_3_ film (Figure 19). During the annealing, TSC was linked to the CsSnI_3_ surface and allowed the lower nucleation site to achieve a bigger grain surface. The passivated CsSnI_3_ film with TSC interlayer exhibited a higher efficiency of 8.20%. Such high efficiency with low tolerant CsSnI_3_ was possible due to the suppressed deep defect densities of undercoordinated Sn^2+^ and vacant Sn inhibiting the nonradiative recombination.

#### 2.2.6. Solution Engineering

**Figure 20:**
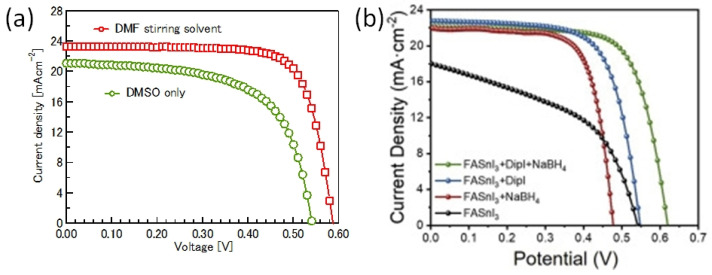
(**a**) Solvent stirring effect on photoelectric performance (24 h). Reprinted with permission [94]. Copyright 2022, American Chemical Society. (**b**) Average representative J–V curves with and without additive after 3 days of light soaking. Reprinted with permission [115]. Copyright 2022, Elsevier Inc.

Sn-PVK precursor solutions are commonly dissolved in DMF:DMSO solvents. In 2019, Baranwal at al first reported that stirring the precursor solution of Sn-PVK in DMSO as cosolvent oxidizes Sn^2+^ into Sn^4+^ [38]. This was confirmed by electrical conductivity and Seebeck coefficient measurements. Later in 2020, Abate et al. found a similar phenomenon using NMR spectroscopy that Sn^2+^ is oxidized under the DMSO effect [100]. Thereafter, different solvents were explored as substituents for DMSO. Using DEF:DMPU as a solvent to dissolve Sn-PVK, an efficiency of 6.2% was reported (Voc = 0.533 V, Jsc = 21.9 mA cm^−2^, FF = 0.53) without using SnF_2_, Sn(0), and reducing agent. The solar cells were fabricated in the inverted structure as PEDOT:PSS/Sn-PVK/C60/BCP/Ag [101]. Subsequently, various efforts were made to limit the use of DMSO as a co-solvent. However, DMSO can slow down the rapid crystallization of Sn-PVK by forming the SnI_2_-3DMSO complex. Thus, DMSO remains an essential co-solvent. Baranwal et. al. investigated the aging effect of the Sn-PVK precursor solution on the photoelectric performance of Sn-PVK solar cells [94]. It was found that DMSO as a co-solvent can increase the oxidation rate of the Sn-PVK solution. Conversely, DMF as a stirring solvent can suppress such Sn-PVK oxidation inside the solution. In addition, GeI_2_ as an additive was shown to control the effect of oxidative solvents. A judicious strategy was followed to fabricate the solar cells. The Sn-PVK precursor solution was continuously stirred in DMF only and a controlled amount of DMSO was added 5 min before the deposition of the Sn-PVK film. In this process, PVK solution stoichiometry was maintained. An efficiency of 10.26% was obtained when DMF was used as the stirring solvent and an efficiency of 7.12% was obtained when DMSO was used as the stirring solvent (Figure 20a). This report also showed that the micro-strain of Sn-PVK films fabricated with a 24 h stirring was reduced to 6% compared to the 9% with 2 h of stirring. DMF was used as a solvent to dissolve Sn-PVK, and a controlled amount of DMSO was added 5 min before the film fabrication. Recently, Abate et al. used 4-tert-butyl pyridine as a co-solvent with DMF and 1,3-dimethyl-2-imidazolidinone [102]. DMSO and SnF_2_ were not used in the fabrication process. Inverted structure solar cells with a photoactive layer of FA_0.78_MA_0.2_EDAI_0.02_SnI_3_ achieved an efficiency of 7.2%. Wu et al. showed that the color of SnI_2_ powder dissolved in DMF changed rapidly in air compared to SnI_2_ dissolved in DMSO [103]. This is because SnI_2_ forms a complex with DMSO, which reduces the dissociation of SnI_2_. However, DMSO itself is oxidative, so various co-solvents were used as a co-additive to DMF. The tin perovskite precursor was dissolved in DMF and N-methylformanilide (NMF) or DMF and 4-acetaminophen (AP). Inverted Sn-PVK solar cells with DMF-AP showed an efficiency of 10.03% and DMF-NMF-based solar cells showed an efficiency of 8.68%. Diau et al. investigated the two-step fabrication of Sn-PVK [104]. In the first step, SnI_2_ and SnF_2_ were coated with DMSO as the solvent. In the second step, FAI and EDAI_2_ were coated with a solvent system hexafluoro-2-propanol (HFP), isopropanol (IPA), and chlorobenzene (CB). The strong interaction of SnI_2_ with IPA inhibited perovskite formation, so, HFP was used to suppress the reactivity of IPA by establishing hydrogen bonding with IPA. The CB used in the second step served as an antisolvent. 

#### 2.2.7. Additive Technology

GeI_2_ was used as an additive in the fabrication of Sn-PVK solar cells to reduce defects and control the back-ground charge density. Small radius Ge can fill the voids in the crystal as well as the surface holes to minimize the defects and vacancies. The sacrificial oxidation of Ge forms a GeO_x_ layer at the top and bottom surface of Sn-PVK, which serves as a protective layer against moisture and humidity ingress. Ge can also sit at the grain boundary [95,105,106]. EDAI_2_ and BAI were used as additives in Sn-PVK solar cells [93]. BAI changed the surface morphology to fuse the crystal boundary which significantly improved the crystallinity and controlled the crystal growth direction. EDAI_2_ curbed the fast crystallization and passivated the defects of Sn-PVK film by controlling the Sn^2+^ vacancy defects and Sn defects. These two additives act together to improve the photoelectric performance of Sn-PVK solar cells. Their role is discussed in detail in the additive engineering section. Phenylhydrazine chloride (PHCl) was used as an additive in FASnI_3_. The phenyl group is hydrophobic, and has 2D nature and hydrazine is a reducing agent. Bulky 2D cations are insulating and impede the photoelectric properties, therefore 3D/2D or 3D/quasi2D photoactive layers are fabricated. PHCl as an additive suppressed Sn^4+^ defects and repair Sn vacancies making the bigger crystal size. The bulky phenyl moiety was incorporated into the crystal lattice, blocking oxygen and H_2_O. The addition of chloride salt makes the VBM and CBM deeper, which allows a better energetic alignment with PEDOT:PSS and C60. An efficiency of 11.4% was obtained showing stable behavior when stored in the glove box for 110 days. However, the solar cells showed decreasing efficiency when exposed to air. When these cells were stored in a glove box, the initial efficiency was recovered under the hydrophobic and reductive effect of PHCl. An enhanced 24.2 ns lifetime was reported compared to 7.6 ns of the pristine film [107]. It is worth noting that, hydrazinium chloride, hydrazinium iodide, and hydrazine vapor have been already utilized as additives and reducing agents to boost the photoelectric performance in Sn-PVK solar cells [108,109,110]. Zou et al. explored the combined beneficial effect of benzylamine and the F atom by using 4-fluorobenzylammonium iodide (FBZAI) as an additive to FASnI_3_ [51]. The benzylamine and the F atom served a dual purpose; benzylamine is hydrophobic, has π conjugated system, and shows conducting behavior; conversely, fluorine has a high electronegativity to tune the electron density. Enhanced spectra in the region of 450–650 nm showed reduced defect density in the bulk, and improved spectra in 750–850 nm showed better contact at the PVK/C60 interface, suggesting fewer traps at the interfaces. Thus, FBZAI also improved the quality of the C60-Sn-PVK interface by reducing the interface defect states, resulting in easy charge transfer. Due to the high electronegativity of F, FBZA^+^ attracts electrons from adjacent atoms to become more negatively charged and NH_3_^+^ becomes more positively charged. Such a dipole synergistically passivated the defect states by forming ionic or hydrogen bonds. An increased efficiency of 13.85% was reported. A similar mechanism of defect passivation was earlier employed using additives benzyl amine, fluoro-phenethylammonium iodide, and pentafluorophen-oxyethylammonium iodide [111,112,113,114]. Ivan et al. reported FASnI_3_-based solar cells with dipropyl ammonium iodide and sodium borohydride as dual additives [115]. Di-propyl ammonium is a bulky 2D ion, whereas sodium borohydride is a reducing agent. The synergetic effect of these two additives achieved a smooth and defect-tolerant film. After three days of light exposure, the solar cells achieved 10.80% efficiency (Figure 20b). The fabricated solar cells showed maximum power point stability for 1300 h measured in an N_2_ atmosphere with a UV filter. The reduced Sn^+2^/Sn^+4^ and I^−^/I_2_ ratios observed in the XPS measurements compared to the pure FASnI_3_ were attributed to the ability of the additives to passivate and limit the loss of iodine species.

**Figure 21:**
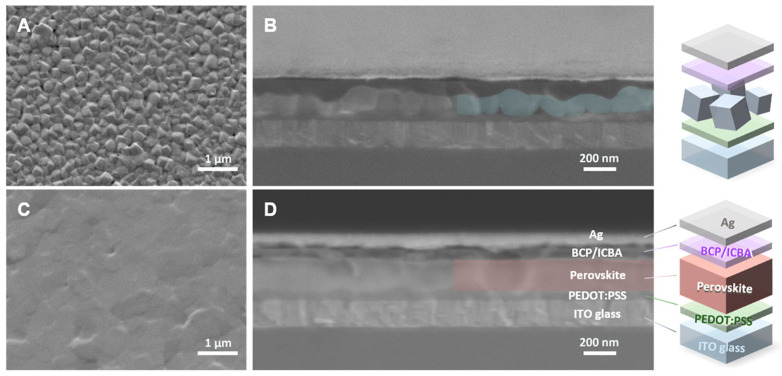
Pristine Sn-PVK (**A**) surface morphology, (**B**) cross-section, Trimethyl urea assisted Sn-PVK (**C**) surface morphology, (**D**) cross-section. Reprinted with permission [51]. Copyright 2022, American Chemical Society.

Zhao et al. used ethylenediammonium halide salts EDAI_2_ and EDABr_2_ as additives in the fabrication of Sn-PVK solar cells [50]. Although both salts were able to suppress oxidation and passivate trap states, EDABr_2_ was more effective in passivating the grain defects, reducing the Sn vacancies, and controlling the background charge density. This was possible because Br^−^ with its small radius has a larger electrostatic potential and shorter bond length between Sn and Br than between Sn and I. This strategy leads to the suppression of deep $Sn_{I}$ defect states. A high efficiency of 14.23% was reported. Chen et al. used 3,3′-(((2,2-diphenylethene-1,1-diyl)bis(4,1-phenylene))bis(oxy))bis(N,N-dimethylpropan-1-amine)tetraphenylethene] (PTN-Br) as an additive to FASnI_3_ [116]. PTN-Br is a π-conjugated semiconducting polymer with deeper VBM. Grain boundaries are a vulnerable site of degradation against UV exposure and moisture/oxygen, and a weaker site for charge conduction. PTN-Br filled the grain boundaries, served as a hole transport center, and improved hole collection, improving the Voc and FF. Dimethylamine of PTN-Br made a Lewis adduct with undercoordinated Sn. The reduced defect states and improved hole collection led to an improvement in efficiency from the original 5.12% (Voc = 0.435) to 7.94% (Voc = 0.544). This report demonstrated that the cells maintained 66% of initial efficiency following 5 h continuous UV exposure due to the protective and coordinating ability of PTN-Br. 2,2,2-trifluoroethylamine hydrochloride (TFEACl) was used as an additive. Bulky TFEA^+^ were present at the grain boundaries, inhibiting the oxygen ingress and Cl^−^ was inserted into the lattice, extending the crystallinity. The defect states were suppressed and improved efficiency was reported [117]. Mi et al. synthesize a 0.8M solution of SnI_2_ by dissolving Sn granules and I_2_ in a solvent mixture of DMF:DMSO (4:1 *v/v*) [51]. This simple method yielded a clear yellow solution of SnI_2_ with overnight dissolution. After this, FAI, PEAI, and trimethyl thiourea were dissolved in SnI_2_ solution to yield Sn-PVK precursor for solar cell fabrication. Thiourea worked as a soft Lewis base additive and the functional group C=S had a greater affinity towards Sn^2+^, the N-H group of thiourea formed a hydrogen-bond donor to I^−^ defects, resulting in a uniform and compact layer with fewer Sn^4+^ defects. Thiourea and the PEDOT:PSS formed hydrogen bonding. This strategy had multiple advantages of being flat, compact with great adhering, and overlapped grain boundary with greater crystal size FASnI_3_. The cells were able to achieve a record efficiency of 14.3% with 0.92V Voc. Moreover, such a facile SnI_2_ preparation process shows that extremely pure SnI_2_ granules are not essential to attain high photoelectric performance. [87] Figure 21 shows the cross-section and surface morphology of the fabricated films. Trimethyl urea as an additive lead to the fusion of grain boundaries to suppress the defects and achieve high efficiency. This report also showed a facile method of fabrication of the SnI_2_ solution and a method of cost-cutting, as SnI_2_ beads are costlier than PbI_2_. Polyvinyl alcohol was added to the FASnI_3_ precursor solution. The hydrogen bonds between the hydroxyl group and iodine led to introduce nucleation sites and slowed down the crystallization rate. The migration of iodide ions was suppressed and the overall trap density was reduced. This additive strategy led to improve efficiency and Voc [118]. Nitrogen-doped graphene oxide (N_0.12_GO) was synthesized using GO and urea (NH_2_CONH_2_) [119]. This (N_0.12_GO) was used as an additive in PEDOT:PSS, and Sn-PVK photoactive layer, and an interlayer of Al_2_O_3_- N_0.12_GO was inserted between HTL and Sn-PVK. This strategy improved the grain size and suppressed the defect states of the photoactive layer. The interface charge recombination was prohibited using the Al_2_O_3_-N_0.12_GO interface. However, the N_0.12_GO doped PEDOT:PSS had a better energetic cascade. This method achieved an efficiency of more than 13%**.** In another report, the C60 derivative C60Cl_6_ was used as an additive to regulate the crystallization process of Sn-PVK [120]. The Cl of C60Cl_6_ had an interaction with Sn^2+^, inhibiting the defects by regulating the crystallization process. The electron-deficient C60 cage interacts with the I^−^, passivates the negatively charged defects such as Sn-I antisite, and prohibits ion migration. C60Cl_6_ sits at the grains to stitch the grain and surface boundaries. All these effects suppressed Sn^2+^/Sn^4+^ oxidation to control the defect density and resulted in an efficiency of over 13%. A MABr-alloyed FASnI_3_ film was incorporated into inverted solar cells as a photo-harvesting layer [121]. MABr causes the crystal to grow in a preferential (001) direction imparting improved crystallinity and better charge conduction. Overall, an improved photovoltaic performance of 9.31% efficiency was observed compared to the 7.82% efficiency of the control film.

#### 2.2.8. Three-Layer Mesoscopic Solar Cell

Three-layer mesoscopic PVK solar cells are known for their unparalleled stability among PVK solar cells [122]. Diau et al. fabricated Sn-PVK solar cells based on FTO glass/TiO_2_/Al_2_O_3_/carbon-infiltrated Sn-PVK [123]. In the photoactive FASnI_3_ layer used, I varied with tetrafluoroborate (BF_4_) to form FASnI_3-x_(BF_4_)_x_. The BF_4_^-^ ion had stronger coupling with Sn^2+^ ion, thus, the optimized FASnI(BF_4_)_2_ precursor suppressed Sn^4+^ formation and achieved a maximum 1.3% efficiency with a Jsc of 20.2mA/cm^2^, Voc of 0.194V and FF of 0.34. It should be noted that such poor efficiency may have arisen due to the electron acceptor defect states of TiO_2_ and Al_2_O_3_ [40]. The cells showed excellent dark storage stability at 65% humidity measured over 1000 h. An additive strategy was proposed for the CsSnI_3_-infiltrated three-layer mesoscopic solar cells c-TiO_2_/m-TiO_2_/Al_2_O_3_/NiO/carbon [124]. 2-aminopyrazine was used as an additive to SnF_2_. The pyrazine ring of APZ provides electrons and acts as a Lewis donor to stabilize Sn^2+^. An efficiency of 5.12% was achieved with 0.40 V Voc, 21.7 mA/cm^2^, and 0.59 FF. The solar cells were able to maintain 90% of their original efficiency after being stored in a glove box filled with N_2_ for 100 h. 

#### 2.2.9. ETL/HTL Modification

Chen et al. modified the PEDOT:PSS surface by treating it with tetrafluoro-tetracyanoquinodimethane (F4TCNQ) [125]. The energy mismatch of PEDOT:PSS with Sn-PVK was resolved, as the PEDOT:PSS treated with F4TCNQ showed deeper energetic alignment. C=N group and F^−^ of F4TCNQ were coordinated to Sn^2+^ and halide ions. This coordination prohibited the defect density and passivated the PVK film. The proper energy cascade and reduced defect states at the HTL/Sn-PVK interface led to a jump in efficiency. An electron transport layer (6,6)-phenyl-C61-butyric acid hexyl ester (PCBH) with a flexible hexyl group was used to modify the charge collection at the ETL [126]. The hexyl group was able to enhance the interconnection with Sn-PVK and fullerene resulting in an improved photovoltaic performance of 9.21%. Chen et al. applied monolayer engineering to treat the PEDOT:PSS HTL layer [127]. PEDOT:PSS was treated with a 2PACz monolayer. This resulted in an improvement in conductivity due to the coupling of the O^-^ of 2PACz to the S^+^ of PEDOT:PSS. This anchoring resulted in the VBM of PEDOT:PSS-2PACz being −5.73 eV, which was deeper than the −5.10 eV of PEDOT:PSS. The modified PEDOT:PSS-2PACz bilayer exhibited an improved mobility of 18.45 compared to the 8.85 cm^2^/V·s of the original PEDOT:PSS. FASnI_2_Br had a VBM of 5.82 eV. Better energetic and crystallization properties led to an improved efficiency of 8.66% for the Sn-PVK solar cells with a bandgap of 1.66 eV. Nitrogen-doped graphene oxide (N_0.12_GO) as an additive in PEDOT:PSS as HTL showed deeper work function and better energetic matching leading to improved Voc in the operation of Sn-PVK solar cells. An interlayer of Al_2_O_3_-N_0.12_GO on PEDOT:PSS- N_0.12_GO as HTL could suppress the interface defects, resulting in a high Voc of 0.961 V [119].

#### 2.2.10. Flexible FASnI_3_ Solar Cell

**Figure 22:**
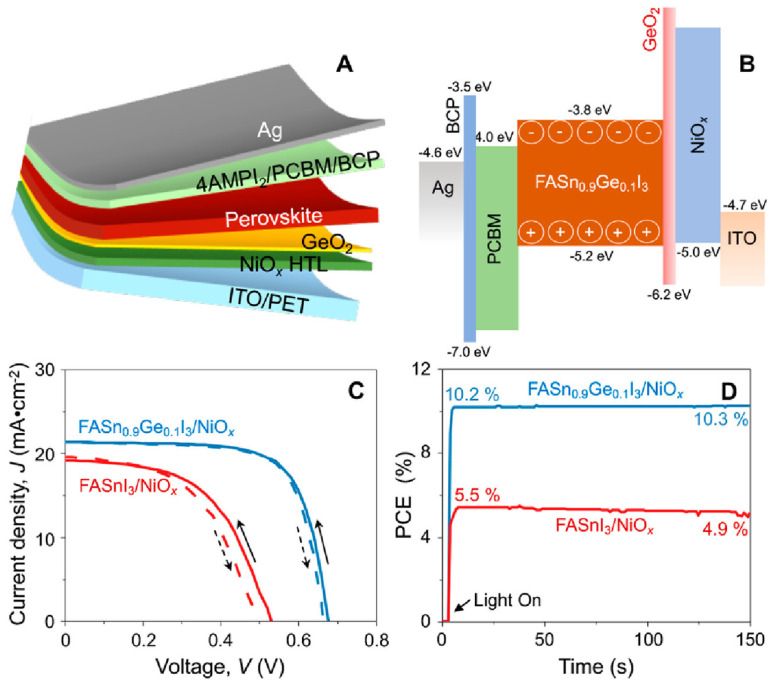
(**A**) Schematic of flexible Sn–PVK solar cells, (**B**) Energy diagram, (**C**) Best J–V characteristics with and without GeO_2_, (**D**) MPPT tracking. Reproduced with permission [77]. Copyright 2022, American Chemical Society.

Portable FASnI_3_ solar cells were realized by the additive strategy of graphite phase C_3_N_4_ (g-C_3_N_4_) [128]. g-C_3_N_4_ follows the size-effect to retard the crystallization rates by providing a crystallographic template and passivating the defect states. The hydrogen bonding between FA and g-C_3_N_4_ optimized the lattice matching and passivation on a flexible substrate. The 2D nature of g-C_3_N_4_ helped to improve the stability of the solar cells. A respectable efficiency of 8.56% was achieved in inverted structure solar cells based on a flexible polydimethylsiloxane substrate. The flexible solar cells maintained 92% of their original efficiency for 300 cycles at a 3 mm curvature radius. Padture et al. fabricated FASnI_3_-based flexible solar cells by Ge alloying (Figure 22) [77]. An amorphous GeO_2_ was formed on the NiO_x_-HTL layer to serve as a barrier to the Sn^2+^ oxidation and allow better bonding between the Sn-PVK and NiO_x_ layers. The Sn-PVKs were coated with 4-(aminomethyl) piperidinium diiodide. A record efficiency of 10.43% was obtained for the flexible solar cells. The cells showed near-stable and consistent performance under 700 h of illumination in a glove box filled with N_2_. The cells were also stable in the 5 mm radius banding test for 1000 cycles.

#### 2.2.11. Electrode Engineering

Han et al. proposed Sn-PVK solar cells without a hole transport layer fabricated on ITO glass [129]. They showed that the addition of EDAI_2_ was essential to achieve better band bending and charge extraction in the case of HTL-free Sn-PVK solar cells. Such solar cells achieved a remarkable efficiency of over 10%. The fabricated HTL-free solar cells showed high stability. They retained 90% of the original efficiency after a heat test at 80 °C for 300 h in a N_2_ medium. Diau et al. found that continuous photo illumination increases the temperature of solar cells and corrodes the Ag electrodes [80]. Thus, one of the stumbling issues with the stability of the solar cells was the corrosion of the Ag electrodes. Hatton et al. investigated the test of Sn-PVK solar cells soaked in ambient light [130]. They found that the combination of bathocuproine and copper electrodes can prevent the corrosion effect of I_2_ (which is formed during the decomposition of Sn-PVK). In addition, the copper electrodes are more resistant to the ingress of water and O_2_. However, the use of copper electrodes has the practical limitation that the energetics does not match the Sn-PVK photoactive layer, which is a limitation that must be tackled. 

The photoelectric parameters of recently published efficient Sn-PVK solar cells with >10% efficiency are summarized in Table 5. The table also shows that the Voc loss, which is mainly caused by nonradiative recombination, should be further lowered because this Voc loss is much higher than Pb-counterparts [131]. 

## 3. Thermoelectric Devices 

In industrial or domestic applications, a major part of the primary energy used is lost in the form of heat, known as waste heat. This waste heat can be converted into electrical energy using thermoelectric devices. 

**Figure 23:**
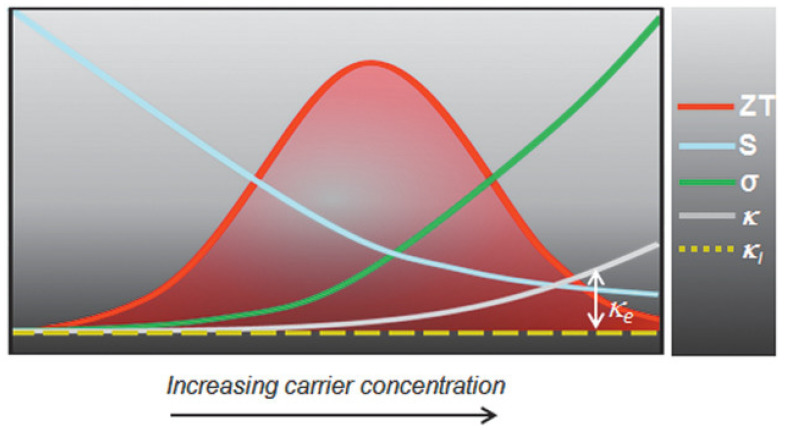
Schematic representation of the interrelationship and trade-off among various thermoelectric parameters. Reprinted with permission under a creative commons license [2]. Copyright 2020, Wiley-VCH.

The performance of such devices is determined by a dimension-less term, the figure of merit $ZT=\left(\sigma S^{2}/k\right)T$, where σ is the electrical conductivity, S is the Seebeck coefficient, k is the thermal conductivity, and T is the temperature. The ability of thermoelectric film to generate power is determined by the term, the power factor $\sigma S^{2}$. Figure 23 shows the relationship between charge carrier concentration n,σ,S, and k. It is clear that n,σ,S, and k are interdependent, and thermoelectric devices can achieve optimum performance with moderately high carrier density or electrical conductivity. If the carrier density is too high, or too low, thermoelectric performance can be compromised. Electrical conductivity is defined as $\sigma =n\mu e=\frac{ne^{2}\tau }{m^{*}}$, where n is the carrier concentration, μ is the mobility, e is the electronic charge, τ is the relaxation time, and $m^{*\;}$is the effective mass. The electrical conductivity can be controlled by varying the charge carrier concentration or mobility. Perovskite crystals are prepared at low temperatures. Such low-temperature fabrication allows them to have many defects. These defects are n-type or p-type. Therefore, the charge carrier density of the perovskite material can be controlled by manipulating the defect density. The surface morphology influences mobility. Rough surfaces with smaller grains, dopants, larger grain boundaries, and disordered energy landscapes have lower mobility. A higher unit cell volume leads to a high charge carrier density, and a higher crystal size leads to a lower charge density. Electrical conductivity can be measured using the four-probe method or I-V characteristics. Temperature-dependent measurements of electrical conductivity predict whether the material behaves as a semiconductor or a metal. Depending on whether the majority of the charge carriers are electrons or holes, the materials can be classified as n-type or p-type.

**Figure 24:**
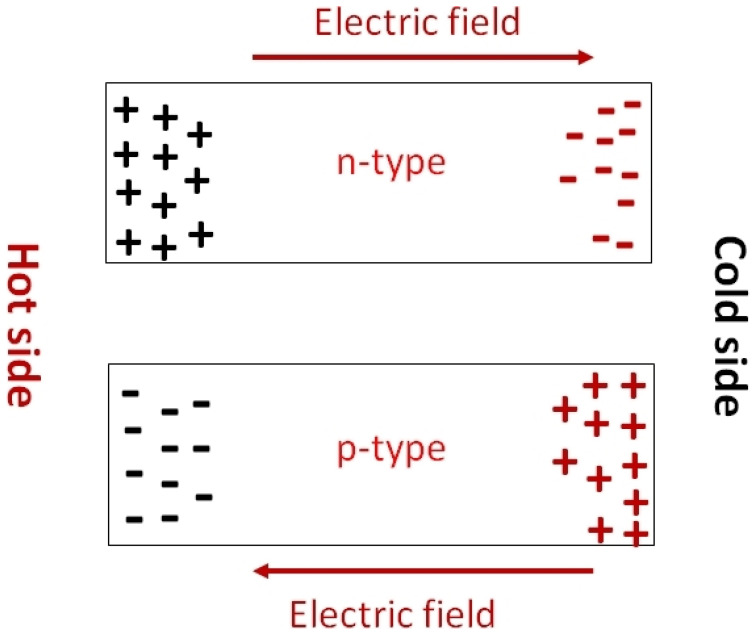
Schematic representation of the charge carrier motion under temperature gradient showing that the Seebeck effect can determine whether the conduction is p- or n-type.

The Seebeck coefficient determines the potential difference that occurs under the influence of a temperature difference. It is defined as S=∇V/∇T. When the temperature difference is applied, a temperature gradient is created at the substrate. The hot region of the substrates contains most of the charge carriers at high temperatures. These charge carriers move toward the lower temperature region, under the influence of the temperature gradient. Depending on the nature of the majority charge carriers of the semiconductor, the charge distribution will take the opposite direction (Figure 24). Thus, Seebeck coefficient measurements also determine whether the substrates are p-type or n-type. P-type and n-type materials have a positive and negative value of S respectively. The measurement of the Seebeck coefficient of unipolar materials with a distant E-E_f_ is determined by Equation (4) [58].

(4) $$S=\frac{k_{B}}{e}\left[\left((E_{f}-E_{trans})/k_{B}T-A\right)\right]$$

where $k_{B}$ is the Boltzmann constant, $E_{f}$ is the Fermi energy level, $E_{trans}$ is the transport energy, and ***A*** is the heat of transport. Equation (4) shows that, as the conductivity increases, the Fermi level gets closer to minimizing $E_{f}-E_{trans}$, as $E_{f}=E_{trans}$, which reduces the Seebeck coefficient. It also shows that p- and n-type semiconductors will have a positive and a negative value of S. However, for $E=E_{f}$, A becomes the dominant factor, and S is determined by the Mott relation shown in Equation (5) [58].

(5) $$S\approx A\approx \frac{\pi^{2}k_{B}^{2}T}{3e}{\left(\frac{d\left(ln\sigma \right)}{dE}\right)}_{E_{F}}=\frac{\pi^{2}k_{B}^{2}T}{3e}{\left(\frac{1}{N}\cdot \frac{dN\left(E\right)}{dE}+\frac{1}{\mu }\cdot \frac{d\mu \left(E\right)}{dE}\right)}_{E_{F}}$$

where σ, E, and N are the electrical conductivity, energy, and density of state, respectively, and $N\left(E\right)$ and $\mu \left(E\right)$ are the density of state/carrier density and mobility at energy E**,** respectively. For metals and degenerate semiconductors, with energy-independent scattering approximation and parabolic band, the Seebeck coefficient is given by

(6) S=4π2kB2eh2m∗T4π3n23 

The thermal conductivity $\left(K\right)$ is defined as

(7) $$K=K_{l}+K_{e}=K_{l}+\sigma LT\;$$

where $k_{l}$ is the lattice component of thermal conductivity, which is determined by the phonon motion resistance within the lattice crystal, and $k_{e\;}$is the electronic part of thermal conductivity, which is determined by the Wiedemann–Franz law. It correlates electronic conductivity with the electronic component of thermal conductivity. The value of the Lorentz number $\left(L\right)$ is always a challenge for new thermoelectric materials. For metals, $L=2.54\times 10^{-8}W\Omega K^{-2}$ [138].

To improve the figure of merit, a reduction in thermal conductivity is required. The reduction in $k_{l}\;$can be achieved by partially decoupling S and k so that the phonon scattering is stronger than the electron scattering. This can be achieved by including the loosely bound atoms inside the crystal as rattling modes. Sn-PVKs and Pb-PVKs have such a rattling mode. CsSnI_3_ has a cluster rattling motion of the small radius Cs atom and the Cs-I bond. Due to this rattling motion, the mean free path lengths of phonons are only 3.9 nm [21]. Another way to lower $k_{l}\;$is to introduce defect states at the atomic level or to create grain boundaries. It has been shown that the electronic thermal conductivity can be neglected for most semiconductors because it is relatively small compared to the thermal conductivity of the lattice. CsSnI_3_ (nanowire) has a thermal conductivity of 0.38 W/m·K at 300 K [21]. This extremely low thermal conductivity is equivalent to phonon glass crystals, an ideal material for heat storage. 

Sn-PVK and Pb-PVK crystals are soft materials. Their electronic properties can be tuned and changed by varying the fabrication protocol. After their successful use in solar cells, Pb-PVKs were explored as thermoelectric films. Due to low electrical conductivity (10^−7^ S/cm), despite a high Seebeck coefficient (6500 S/cm) and low thermal conductivity values (0.32 W/m·K), poor thermoelectric performance was reported [139]. Sn-PVKs have the advantage of relatively high carrier concentration, which leads to high electrical conductivity. It is worth noting that highly conductive Sn-PVKs are feasible due to the presence of Sn^4+^, which has a metallic character. However, Sn^4+^ can be formed by the oxidation of Sn^2+^. Such oxidation creates an Sn^2+^ vacancy and eliminates alternative octahedra from the Sn-PVK crystal [42]. Thus, Sn-PVK as a thermoelectric film has two contradictory positive consequences, (i) The oxidation of Sn^2+^ to Sn^4+^ contributes to high electrical conductivity, and (ii) The oxidation of Sn^2+^ eliminates the alternating octahedra, resulting in a decrease in phononic thermal conductivity. 

These two unusual phenomena act synchronously to improve the figure of merit. Therefore, the high electrical conductivity, respectable Seebeck coefficient, and extremely low thermal conductivity make them attractive for thermoelectric applications. However, among Sn-PVKs, CsSnI_3_ exhibits high electrical conductivity and low effective mass [21,32,33,34]. Therefore, CsSnI_3_ is the most widely used Sn-PVK candidate in the field of waste heat recovery. Such solution-manufactured, low-temperature thermoelectric films have the potential to be used for the Internet of Things, and wearable devices [140].

When a substrate has maintained a temperature gradient, a potential difference is generated. The generation of this electrical energy depends on various thermoelectric parameters (S, σ, and k.). Optimization of these parameters is attempted in Sn-PVK through additive, substitution, passivation, and nanocomposite structures. These developments are discussed in detail below.

### 3.1. CsSnI_3_ Thermoelectric Film 

**Figure 25:**
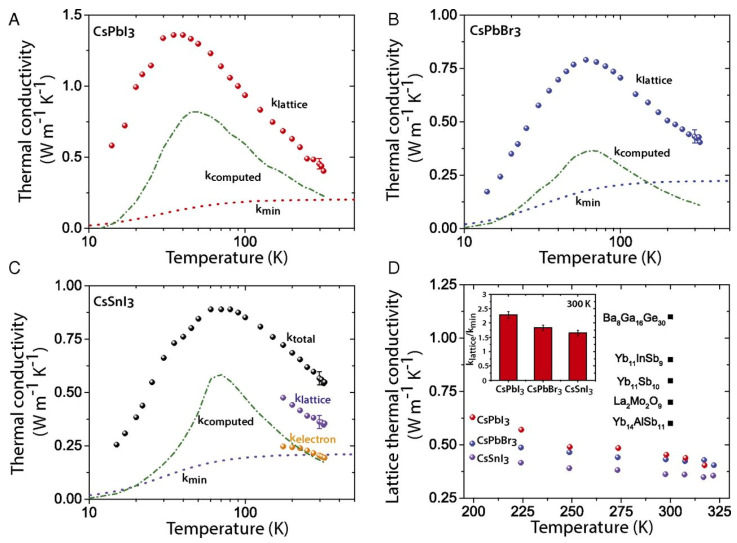
Thermal conductivity of all inorganic perovskite. Measured thermal conductivity (**A**–**C**) K_lattice_ A, and B, K_total_ in C. CsSnI_3_ thermal conductivity is calculated from observed conductivity and Wiedemann–Franz law. K_total_ = K_lattice_ + K_electron_. (**D**) Comparison with other inorganic single crystal and inorganic materials having ultra-low thermal conductivity. Reproduced with permission [21]. Copyright 2017, National academy of Sciences.

**Figure 26:**
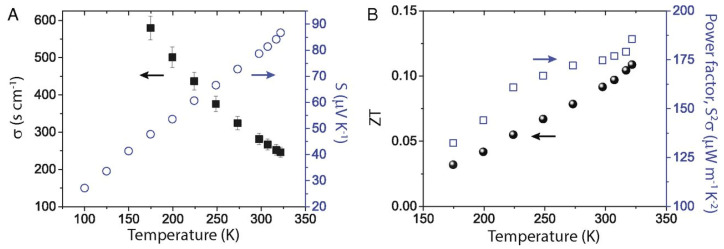
(**A**) Electrical conductivity and Seebeck coefficient variation with temperature, (**B**) Power factor and figure of merit variation with temperature. Reproduced with permission [21]. Copyright 2017, National academy of Sciences.

Lee et. al. synthesized CsSnI_3_ nanowires for thermoelectric applications using a two-step process [21]. In the first step, CsI was deposited and then SnI_2_ was deposited in the second step. The fabricated single crystalline CsSnI_3_ exhibited extremely low thermal conductivity of 0.38 Wm^−1^K^−1^, the high electrical conductivity of 282 S/cm, and high mobility of 394 cm^2^/V·s at room temperature (Figure 25). They calculated the thermal conductivity of all inorganic CsPbBr_3_ and CsSnI_3_, as shown in Figure 25B,C. These values are extremely low compared to existing common thermoelectric materials such as Ba_8_Ga_16_Ge_30_, and Yb_14_AlSb_11_. However, the thermal conductivity of CsSnI_3_ is lower than the other all-inorganic perovskite counterparts, CsPbI_3_ and CsPbBr_3_. The obtained thermal conductivity was extremely low, comparable to an amorphous limit value, and other existing inorganic thermoelectric materials (Figure 25D). The reason for this extremely low thermal conductivity was explained by the cluster rattling of various crystal atoms and the Cs-I bonding. The measurement of temperature-dependent electrical conductivity confirmed the metallic behavior of CsSnI_3_ (Figure 26A), and the Seebeck coefficient measurements showed the opposite pattern of electrical conductivity. The measured ZT and power factor are shown in Figure 26B. This report highlighted an important step for the use of CsSnI_3_ as a thermal-to-electrical energy converter, highly conductive CsSnI_3_ can be explored as an electrically conductive thermal insulator.

**Figure 27:**
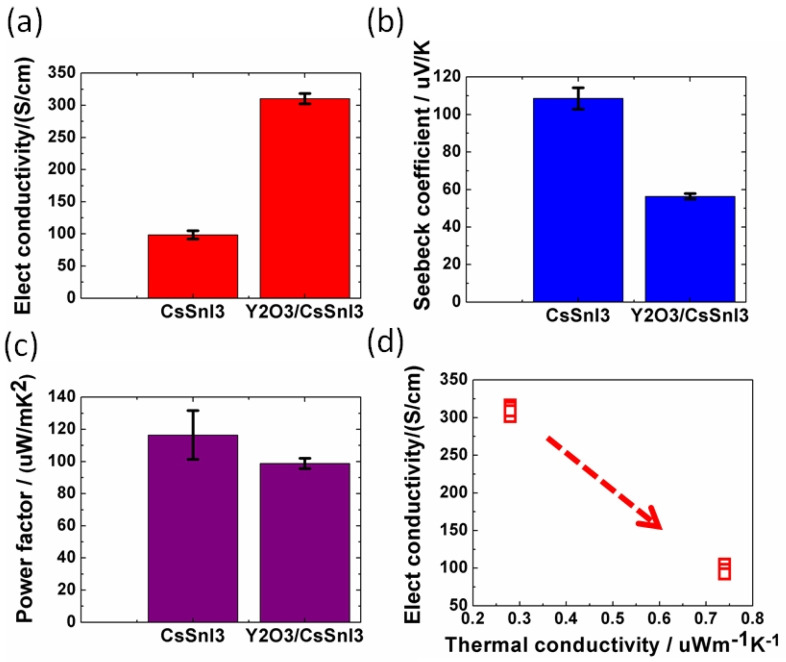
(**a**) Electrical conductivity variation CsSnI_3_ vs. CsSnI_3_/Y_2_O_3_, (**b**) Seebeck coefficient variation CsSnI_3_ vs. CsSnI_3_/Y_2_O_3_, (**c**) Power factor variation CsSnI_3_ vs. CsSnI_3_/Y_2_O_3_ (**d**) Thermal conductivity variation CsSnI_3_ vs. CsSnI_3_/Y_2_O_3_. Reproduced with permission [76]. Copyright 2020, Elsevier.

Saini et al. synthesized CsSnI_3_ thin films using a one-step coating process assisted with the antisolvent dripping method [141]. The spin-coated thin films were heat treated at different temperatures of 50, 70, 100, 130, and 150 °C. The best performance was obtained at 130 °C/5 min annealing treatment. When measured at room temperature (300 K), a ZT = 0.137 was obtained. Later, a mini-module using all inorganic CsSnI_3_ was demonstrated [142]. A decent output power of 0.8 nW was reported and measured at room temperature (300 K). The temperature difference at the hot and cold edges was kept at 5 °C. The Sn-PVK crystals have the advantage of high electrical conductivity and extremely low thermal conductivity. Inspired by the poor photoelectric performance of n-i-p solar cells, a nanocomposite CsSnI_3_/Y_2_O_3_ was fabricated as a thermoelectric layer. This nanocomposite enabled improved electrical conductivity and lower thermal conductivity, resulting in an improved ZT of 0.11 at 300 K. The formation of Sn^4+^ in the nanocomposite CsSnI_3_/Y_2_O_3_ leads to improved electrical conductivity, reduced Seebeck coefficients, and Power factor (Figure 27a–c). The formed Sn^4+^ destroys alternating octahedra of the CsSnI_3_ crystal. These vacancies increase the phonon scattering path and decrease the thermal conductivity (Figure 27d) [76].

**Figure 28:**
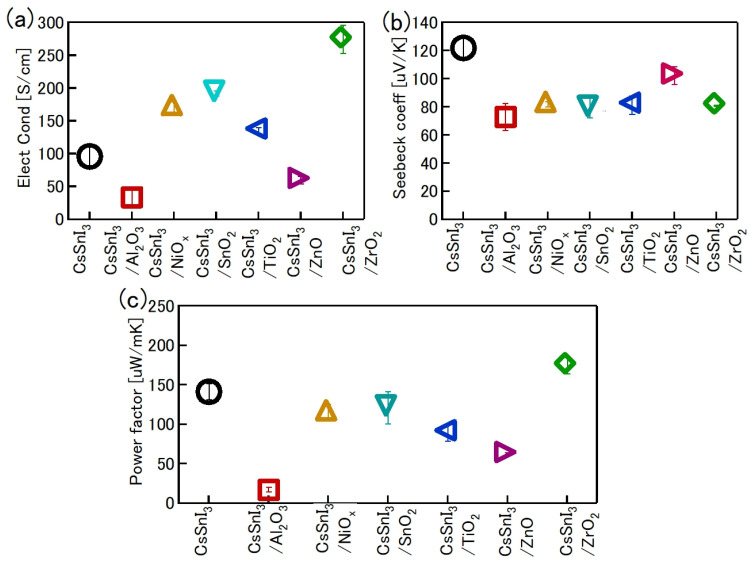
(**a**) Electrical conductivity variation with various CsSnI_3_/metal oxide nanocomposites, (**b**) Seebeck coefficient variation with various CsSnI_3_/metal oxide nanocomposites, (**c**) Power factor variation with various CsSnI_3_/metal oxide nanocomposite. Reproduced with permission [40]. Copyright 2022, American Chemical Society.

In addition, various possible nanocomposites CsSnI_3_/Al_2_O_3_, CsSnI_3_/NiO_x_, CsSnI_3_/SnO_2_, CsSnI_3_/TiO_2_, CsSnI_3_/ZnO, CsSnI_3_/ZrO_2_ were prepared for waste heat-to-electricity conversion (Figure 28). Variable electrical conductivity was obtained, which showed that the thermoelectric properties have a dependency on the inorganic layer. As the electrical conductivity increased, Sn^4+^ also increased linearly (Figure 6a). The electrical conductivity and bandgap energy also showed a linear relationship (Figure 6b). Such a relationship was explained by the Burstein–Moss effect. The reason for this variation of electrical conductivity, which is due to the variation of Sn^4+^, was explained from the point of view of the defect states of the inorganic layers [40].

**Figure 29:**
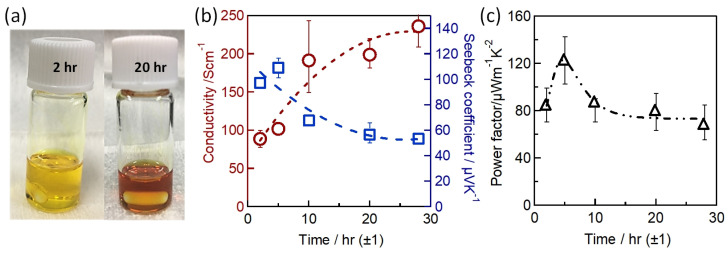
(**a**) CsSnI_3_ precursor color change under DMSO co-solvent following 20 h of stirring at 70 °C hotplates. The vial was stirred inside a glove box (O_2_ and H_2_O < 0.5 ppm), (**b**) Variation in Electrical conductivity and Seebeck coefficient with the precursor stirring time, (**c**) Power factor variation with stirring time effect. Reproduced with permission [38]. Copyright 2019, Springer Nature.

Solution coating is a simple method to prepare perovskite thin films. To get a uniform solution, stirring the precursor is the preferred method. In 2019, Baranwal et al. found, that the stirring time of a Sn-PVK solution can affect its electronic properties (Figure 29) [38]. They controlled the electrical conductivity of the CsSnI_3_ thin film by monitoring the stirring time of the precursor solution. DMSO is a common co-solvent for dissolving Sn-PVK. However, DMSO can initiate the formation of Sn^4+^ during the oxidation of Sn^2+^ in the precursor itself. The formed Sn^4+^ leads to an increase in charge density and thus an increase in electrical conductivity. The formation of Sn^4+^ was confirmed by XPS measurements. The XRD pattern indicates that the peak intensity was suppressed, and the UV-vis absorption spectra showed a blue shift when the stirring time and Sn^2+^ vacancies were increased. Optimal performance with a power factor of 145.10 µWm^−1^K^−2^ was achieved at 5 h stirring.

**Figure 30:**
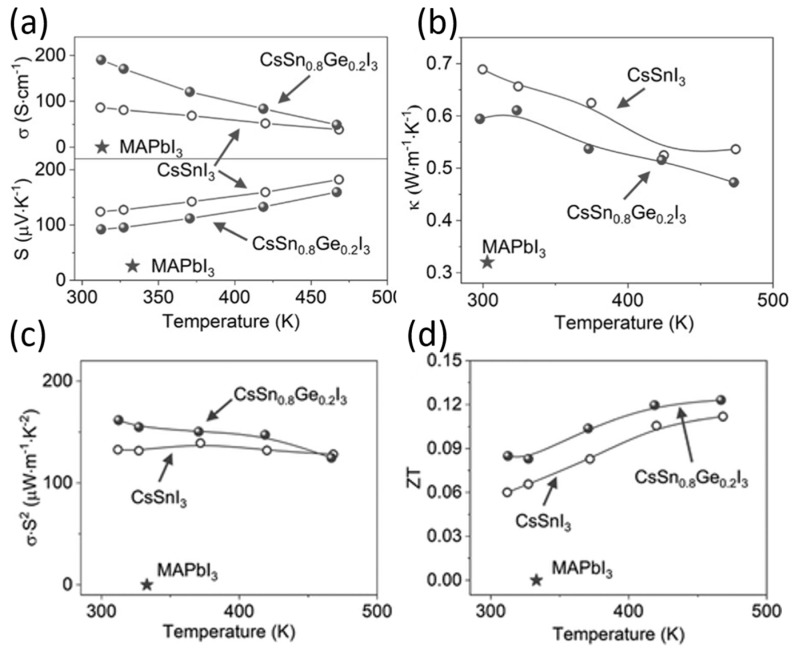
(**a**) The measurement of electrical conductivity and Seebeck coefficient under variable temperature, (**b**) The measurement of thermal conductivity under variable temperature, (**c**) Calculated power factor under variable temperature, (**d**) Calculated figure of merit under variable temperature. Reproduced with permission [144]. Copyright 2020, American Chemical Society.

Fenwick et al. fabricated CsSnI_3_ thin films using two different thermal deposition methods under a high vacuum [143]. Both methods use sequential deposition of CsI and SnI_2_ but differ in order. The stability of films was strongly influenced by the order of deposition. CsSnI_3_ (CSS) films deposited with CsI, then SnI_2_, had poor stability under a N_2_ atmosphere and air. CsSnI_3_ (SCS) film deposited with SnI_2_, then CsI, had better stability under air exposure and N_2_ atmosphere. Temperature-dependent conductivity measurements showed that both CsSnI_3_ films behave as semiconductors. This behavior is the opposite of that reported by Lee et al. [21]. Sn-PVKs are highly oxidative under air exposure. This drawback was exploited to study thermoelectric performance on-air exposure. Upon air exposure, the thermoelectric performance was improved, however, this process has the drawback of reproducibility. Pb-free CsSn_0.8_Ge_0.2_I_3_ alloyed ingots were prepared by vacuum deposition by Padture et al. [144]. Ge substitution leads to a more non-uniform bond distribution of bonds within the lattice, and non-harmonization of the cluster rattle phenomenon. Such a strategy improved the electronic conductivity and decreased the thermal conductivity resulting in a better power factor. Ge-alloyed CsSnI_3_ had improved electrical conductivity, power factor, and figure of merit over a measured temperature range of 300 to 460 K (Figure 30). However, in another report on solar cells, Padture et al. achieved better photoelectric efficiency with Ge substitution [77]. This may be related to the change in the fabrication process, as Ge^2+^ can oxidize to conductive Ge^4+^ to affect the electronic properties of the semiconductor. In addition, the different stoichiometry of GeI_2_ addition in the perovskite solution can change the performance of the perovskite film in different device applications. Kanazatis et al. fabricated CsSnBr_3-x_I_x_ ingots from CsBr, CsI, SnI_2_, and SnBr_2_ powders [145]. They showed that with the stoichiometric increase in I, the electrical conductivity increased, and conversely, the Seebeck coefficient decreased. They also showed that the Cs atom sits at an eccentric position, causing the rattling phenomenon and making the thermal conductivity extremely low. CsSnBr_3_ showed a high Seebeck coefficient of 325 µV/K with an electrical conductivity of 2 S/cm at 300 K. The measurement of temperature-dependent electrical conductivity showed that the conductivity values exhibit a decreasing behavior, i.e., CsSnBr_3−x_I_x_ exhibits metallic behavior. A ZT of 0.15 was determined at 550 K for the CsSnI_3_ ingot.

**Figure 31:**
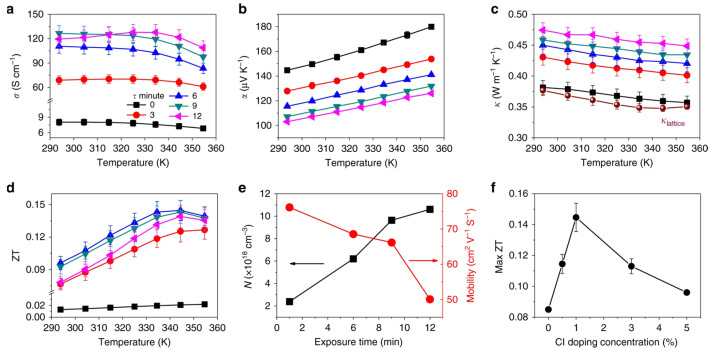
Thermoelectric performance of CsSnI_3-x_Cl_x_ (SnCl_2_ = 1%) thin films (**a**) Temperature dependence on electrical conductivity, (**b**) Temperature dependence on Seebeck coefficient, (**c**) Temperature dependence on thermal conductivity, (**d**) Temperature dependence on the figure of merit, (**e**) Ambient air exposure time influence on charge density and mobility, (**f**) Figure of merit variation with SnCl_2_ substitution. Reproduced from [146] under http://creativecommons.org/licenses/by/4.0/ (accessed on 19 October 2022). Copyright 2019, Springer Nature.

Fenwick et al. have shown that the thin layer of SnCl_2_ over inorganic CsSnI_3_ acts as a sacrificial layer to protect the surface from attack by the ambient medium, while also acting as a dopant for holes [146]. The thermoelectric performance of films was evaluated after 0, 3, 6, 9, and 12 min of air exposure. The films showed increased electrical conductivity and thermal conductivity but a decreased Seebeck coefficient with air exposure time. High thermoelectric performance was obtained with 6- and 9-minute exposure times. The resulting CsSnI_3−x_Cl_x_ film has better thermoelectric performance derived from air exposure with 1% SnCl_2_ (Figure 31).

**Figure 32:**
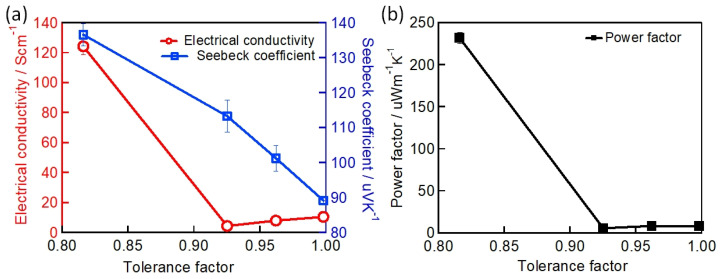
(**a**) Electrical conductivity and Seebeck coefficient variation with tolerance factor. (**b**) Calculated Power factor. Reproduced with permission from [147]. Copyright 2020, the Japan Society of Physics.

The CsSnI_3_ crystal has a low tolerance factor and a high strain. This crystal strain is due to the small radius Cs, which cannot hold the crystal lattice and promotes Sn^2+^ oxidation. In addition, CsSnI_3_ exhibits an exceptionally high charge carrier density among Sn-PVKs. An alloying technique on the A-site was proposed to regulate the perovskite crystal [147]. The large cation FA was substituted at the A-site of CsSnI_3_. As expected, the Cs-FA-mixed Sn-PVK exhibited a better tolerance factor but lower electrical conductivity. One hundred percent and eighty percent FA-based Sn-PVKs have higher conductivity than sixty percent FA-based Sn-PVK. CsSnI_3_, Cs_0.4_FA_0.6_SnI_3_, Cs_0.2_FA_0.8_SnI_3_ and FASnI_3_ have tolerance factor 0.82, 0.93, 0.96 and 0.99 respectively. The Seebeck coefficient showed an opposite trend, and the power factor showed a similar trend to that of the electrical conductivity (Figure 32). This report explains that all inorganic CsSnI_3_ have better thermoelectric performance than FASnI_3_.

### 3.2. FASnI_3_ Thermoelectric Film

Liang et al. used the sequential treatment of air exposure and F4TCNQ surface passivation to improve the hole doping of thermoelectric FASnI_3_ films [148]. Such an air exposure strategy is not a reliable method to control hole doping, as the outdoor humidity and temperature are highly dependent on weather conditions. Depending on the weather conditions, hole doping may slow down or accelerate the doping conditions, and the reproducibility of the results is highly doubtful. The dual strategy of air exposure and coating of F4TCNQ improved the power factor by 10 to 53 µW/m·K^2^. F4TCNQ was fused into the FASnI_3_ film. F4TCNQ^-^ and F4TCNQ^2-^ were formed during electron transfer from FASnI_3_ to F4TCNQ. Gong et al. doped FASnI_3_ with F4TCNQ [149]. The electrical conductivity of FASnI_3_ thin films increased with low doping with F4TCNQ due to better surface morphology and high electrical conductivity. High doping of F4TCNQ in FASnI_3_ decreased the electrical conductivity due to poor surface morphology. F4-TCNQ doped FASnI_3_ thin films exhibit a Seebeck coefficient of ∼310 μVK^−1^, a power factor of ∼130 μW m^−1^ K^−2^ and a ZT value of ∼0.19 at room temperature. The authors report the thermal conductivity of FASnI_3_ to be 0.141 W/m·K at RT.

### 3.3. MASnI_3_ Thermoelectric Film 

MASnI_3_ thin films were investigated for thermoelectric applications using an antisolvent-assisted one-step fabrication method [150]. These films were prepared at various baking temperatures. Films baked for longer time intervals showed increased electrical conductivity due to high Sn^4+^ formation. Measurement of temperature-dependent electrical conductivity showed that MASnI_3_ has semiconducting behavior. Later, the same group studied the effect of antisolvent on thermoelectric performance [151]. The use of antisolvent leads to better cross-linking of the films and the amount of Sn^4+^ was increased. The crystal size of the films prepared by the antisolvent method was increased, resulting in improved electrical conductivity and improved power factor of 1.55 µWm^−1^K^−2^ with a ZT of 0.005 at 320 K. Etgar et al. also showed that the perovskite film becomes more conductive after deposition with antisolvent [152]. Taking advantage of the air-sensitive properties of Sn-PVKs, Baran et al. controlled the thermoelectric performance of MASnI_3_ thin films by aeration [153]. The electrical conductivity of MASnI_3_ and MASn_0.75_Pb_0.25_I_3_ films was increased 1.7–2.4 times by air exposure. The conductivity of MASnI_3_ increased in the first 5 min and then decreased. The conductivity of MASn_0.75_Pb_0.25_I_3_ increased and then decreased, and the Sn^2+^ was converted to Sn^4+^. The air stability of the films was improved by using the p-dopant F4TCNQ. The MASnI_3_ films doped with F4TCNQ were more resistant to the effect of air but the thermoelectric performance was suppressed. 

The degradation product of Sn-PVKs upon air and organic solvent exposure is SnO_2_ and AI (A = Cs, FA, MA) [154]. SnO_2_ is environmentally friendly, however, CsI, FAI, and MAI toxicity are under debate. Following the introduction of European regulations, commercial suppliers have mentioned on their safety data sheet that CsI is more toxic than FAI and MAI. Stoumpous et. al. have reported that the annealing of ASnI_3_ will evaporate Sn^4+^[155]. However, the p-type properties of ASnI_3_ are because of Sn^4+^. Thus, the elimination of Sn^4+^ makes ASnI_3_ an n-type semiconductor, which is beneficial for thermoelectric modules. Considering the degradation products, organic cation-based Sn-PVK may be researched for greater ZT for green energy and a pollution-free society. The thermoelectric performance of Sn-PVK films is summarized in Table 6.

## 4. Challenges and Remedies

**Figure 33:**
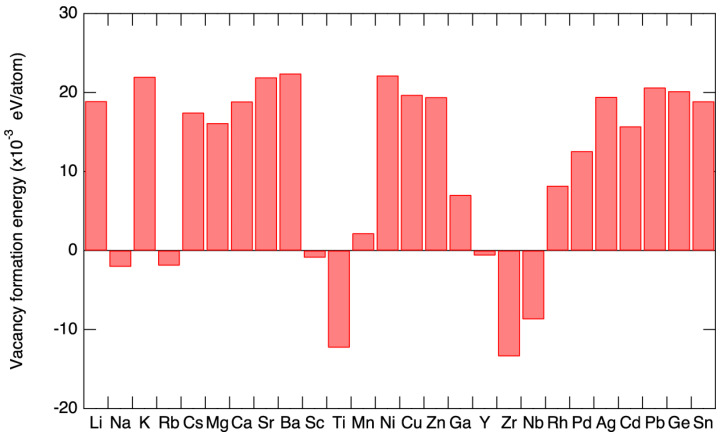
Calculated vacancy formation energy in MASnI_3_ when one Sn atom of the crystal lattice is replaced. Reproduced with permission [157]. Copyright 2022, The Japan Society of Applied Physics.

Sn-PVK active layers tend to generate defective states due to the low formation energy of vacancies. Sn^2+^ are oxidized to form Sn^4+^ defects and Sn vacancies, and Sn^4+^ has metallic conducting properties. This increases the background current density and lowers the charge collection at the interfaces. The primary concern with Sn-PVK layers is the undesired oxidation during fabrication itself. Additional Sn sources in the form of SnF_2_ suppress the formation of vacancies. Inorganic GeI_2_ has also shown a dual role in suppressing the defect states by passivating the vacancies and halide defects and forming sacrificial interfaces at, not only the top layer but the bottom layer, too. Inorganic materials are stable and have prospects for long-term stability. Recently, several inorganic materials have been investigated as possible dopants/substitutes to improve vacancy formation in Sn-PVKs (Figure 33). Among them, Sn doping has already been tried and the rest should be investigated [87]. Low lattice strain and crystal regulation enhance the perovskite stability, and these dopants tend to improve the diffusion length of Sn-PVK. The rapid crystallization of Sn-PVK needs to be addressed by controlling the nucleation and crystallization process. Diau et al. employed BAI and EDAI_2_ to synergistically control the nucleation and growth to reduce the defect states and pinholes [93]. Surface passivation with Lewis bases such as edamine saturates the dangling and unsaturated bonds of Sn-PVK surfaces [84]. These processes act synergistically to balance the energy cascade and minimize charge loss, resulting in increased Jsc and Voc. In addition, the development of the new device structure improved photoelectric performance and stability [49]. A balanced charge transfer is equally important to achieve high photoelectric performance with suppression of bulk defects and interfacial modification. In Sn-PVK solar cells with normal structure, the mesoporous electron and hole transport layer should be passivated to suppress the defects.

However, the complementary application of Sn-PVK is being explored in thermoelectric applications. The extremely low thermal conductivity of Sn-PVK is one of its fascinating properties and electrical conductivity should be further increased using tetra halide salts because excess halides increase the conductivity by forming Sn^4+^, and the created vacancy site and dopants may work as phonon scattering paths to lower the thermal conductivity. However, electrical conductivity can be affected by the use of additives or substitution techniques, and the Seebeck coefficients have a respectable value. Therefore, such Sn-PVK films can be explored simultaneously as photon and thermal waste heat utilization in the form of sensitized Sn-PVK thermal cells [158]. 

## 5. Conclusions

We summarize the recent development of Sn-PVK active layers for photon harvesting and thermal to electric energy harvesting applications. The fundamental properties of Sn-PVK are discussed concerning such wide-ranging applications. Strategies for improving photoelectric and thermoelectric performance are also discussed. We believe, that continued studies on this Pb-free Sn-PVK will open up new dimensions in material physics and allow device engineering to reach new heights by suppressing defect states and improving diffusion length. Simultaneously, Sn-PVK should be explored in sensitized thermal cells. 

## Figures and Tables

**Table 1:** Tolerance factor of tin halide perovskites.

	CsSnI_3_	MASnI_3_	FASnI_3_	FASnI_2_Br	FASnIBr_2_
**Tolerance factor**	0.82	0.93	0.99	1.00	1.01
RCs ^+^ = 167 pm; RMA ^+^ = 216 pm; FA ^+^ = 253 pm; RSn^2 +^ = 115 pm; RI^−^ = 220 pm; RBr^−^ = 196 pm [27]					

**Table 2:** Calculated bandgap energy and effective electron and hole mass ($m_{e}^{*}\;\mathrm{and}\;m_{h}^{*}$ ) of MASnI_3_ and MASnBr_3_. Reproduced with permission [34]. Copyright 2016, Springer Nature.

	MASnI_3_			MASnBr_3_		
	Bandgap/eV	$m_{e}^{*}$	$m_{h}^{*}$	Bandgap/eV	$m_{e}^{*}$	$m_{h}^{*}$
Cubic	0.83	0.17	0.11	1.12	0.19	0.14
Tetragonal Parallel	0.73	0.11	0.10	1.06	0.17	0.14
Tetragonal Vertical	0.93	0.15	0.14	1.36	0.25	0.19
Orthorhombic	1.08	0.19	0.18	1.53	0.22	0.23

**Table 3:** Calculated effective mass ($m^{*}$) at the band edges (without spin-orbit coupling). These values correspond to electron mass. Near VBM, the band is nondegenerate, and the dispersion of the band is isotropic and parabolic (h = hole, e = electron, le = light, he = heavy). Reproduced with permission [33]. Copyrisght 2013, APS Physics.

**Sample**	$m_{h}^{*}\;$	$m_{he\;}^{*}\left[100\right]\;$	$m_{he}^{*}\left[111\right]$	$m_{le}^{*}\left[100\right]$	$m_{le}^{*}\left[111\right]$
**α-CsSnCl_3_**	0.140	0.741	0.272	0.088	0.149
**α-CsSnBr_3_**	0.082	0.635	0.201	0.053	0.084
**α-CsSnI_3_**	0.069	0.573	0.154	0.041	0.068

**Table 4:** The summary of electronic parameters of Sn-PVK layers.

Perovskite	Hole Mobility/cm^2^/V·s	Diffusion Length/nm	Charge Life Time τ [ns]	Acceptor Defect Density [10^15^/cm^3^]	Trap Density [10^15^/cm^3^]	Ref.
(Cs_0.02_(FA_0.9_DEA_0.1_)_0.98_)_0.98_EDA_0.01_I_3_-GeI_2_	_	_	_	_	9.55/SC	[49]
FASnI_3_-GeI_2_-EDABr_2_	2.97 × 10^−2^	_	37.83	5.76/M-S	19.3^/^SC	[50]
FASnI_3_-FPEABr	_	_	2.87	_	3.32/SC	[20]
FASnI_3_-PEAI-trimethylthiourea	_	_	123	_		[51]
FA_0.98_EDA_0.01_SnI_3_	_	_	_	_	4.16/SC	[52]
(FA_x_EA_1-x_)_0.98_EDA_0.01_SnI_3_-GeI_2_	9.27 × 10^−3^	348	20.1	5.48/M-S	29/SC	[53]
FA_0.98_EDA_0.01_SnI_3_/GeI_2_/FBZAI	_	_	19.8	6.76/M-S	_	[54]
FASnI_3_/PEAI/GuaSCN	9 × 10^−4^	_		_	_	[55]
(Cs_0.02_(FA_0.9_DEA_0.1_)_0.98_)EDA_0.01_I_3_)GeI_2_/ACAC/EDA	_	_	12.85	_	9.1 /SC	[56]
FASnI_3_-PEABr/NH_4_SCN/GAA	_	_	3.60	_	9.8 /SC	[57]
CsSnI_2.4_Br_0.6_-dimethyl ketoxime	_	_	15.4	_	12.4/SC	[58]
FA_0.8_GA_0.2_SnI_3_-PHSCN	_		10.8			[59]
(PEA)_0.15_FA_0.85_SnI_2.55_Br_0.45_	_	290				[60]

M-S: Mott–Schottky; SC: Space charge limited current.

**Table 5:** The photoelectric performance summary of Sn-perovskite solar cells with >10% efficiency.

Solar Cell Structure	Efficiency [%]	Jsc [mA/cm^2^]	Voc [V]	FF	Voc Loss [V]	Ref.
ITO/PEDOT:PSS/(FPEA)_0.1_FA_0.9_SnI_2.9_Br_0.1_/ICBA/BCP/Al	14.81	24.91	0.84	0.71	0.59	[20]
ITO/PEDOT:PSS/(PEA)_0.1_5FA_0.85_SnI_2.55_Br_0.45_/ICBA/BCP/Ag	14.63	20.6	0.91	0.77	0.49	[60]
ITO/PEDOT:PSS/FASnI_3_-PEAI-trimethylthiourea/N methyl thiourea/ICBA/BCP/Ag	14.38	20.3	0.92	0.77	0.48	[51]
ITO/PEDOT:PSS/FASnI_3_-GeI_2_-EDABr_2_	14.23	22.49	0.817	0.774	0.563	[47,50]
ITO/PEDOT:PSS/FA_0.98_EDA_0.01_SnI_3_/C60-BPY/C60/BCP/Ag	14.14	23.14	0.82	0.72	0.58	[52]
FTO/P-SnO_x_/(Cs_0.02_(FA_0.9_DEA_0.1_)_0.98_)_0.98_EDA_0.01_I_3_-GeI_2_/T-SnOx/PCBM:ICBA/C60/BCP/Ag	14.09	24.09	0.78	0.75	0.62	[49]
ITO/PEDOT:PSS/FA_0.98_EDA_0.01_SnI_3_/GeI_2_/FBZAI/C60/BCP/Cu	13.85	22.87	0.78	0.78	0.61	[54]
ITO/PEDOT:PSS/FA_0.99_EDA_0.005_SnI_3_/<PMMA>/C60/BCP/Ag	13.82	22.74	0.85	0.72	0.56	[132]
ITO/NiO_x_/FASnI_3_/PEAI/GuaSCN/ICBA/BCP/Ag	13.79	20.32	1.01	0.67	0.39	[55]
ITO/PEDOT:PSS/FASnI_3_-PEABr/NH_4_SCN/GAA/ICBA/BCP/Ag	13.70	19.66	0.93	0.75	0.47	[57]
ITO/PEDOT:PSS/FASnI_3_/EDADI/Sn<6-MaleiMidocaproic Acid Hydrazide, trifluoroacetic Acid>/BCP/C60/Ag	13.64	23.08	0.803	0.73	0.567	[83]
ITO/PEDOT:PSS/FA_0.8_GA_0.2_SnI_3_-(PHSCN)/C60/BCP/Ag	13.50	21.9	0.81	0.76	0.59	[59]
ITO/PEDOT:PSS/FASnI_2.85_Br_0.1_Cl_0.05_/C60/BCP/Ag	13.40	23.02	0.81	0.72	0.59	[133]
ITO/PEDOT:PSS/PEA_0.15_FA_0.85_SnI_3_/C60Cl_6_/C60/BCP/Ag	13.30	20.31	0.86	0.76	0.54	[120]
FTO/PEDOT:PSS-N_0.12_GO/Al_2_O_3_-N_0.12_GO/FA_0.8_MA_0.2_SnI_3_:N_0.12_GO//BCP/Au	13.26	21.21	0.961	0.651	0.42	[119]
FTO/PEDOT:PSS/(FA_x_EA_1-x_)_0.98_EDA_0.01_SnI_3_-GeI_2_/C60/BCP/Ag	13.24	20.32	0.84	0.78	0.56	[53]
FTO/PEDOT:PSS/(Cs_0.02_(FA_0.9_DEA_0.1_)_0.98_)EDA_0.01_I_3_)GeI_2_/<ACAC/EDA>/C60/BCP/Ag	13.00	22.70	0.79	0.72	0.65	[56]
FTO/PEDOT:PSS/FA_0.98_EDA_0.01_SnI_3_/GeI_2_/<Me_3_SiBr>/C60/BCP/Ag	12.22	24.11	0.70	0.72	0.72	[91]
ITO/PEDOT:PSS/FASnI_3_-TG/C60/BCP/Ag	11.78	22.82	0.73	0.72	0.67	[90]
ITO/PEDOT:PSS/FA_0.75_MA_0.25_SnI_3_/TM-DHP/<EDA>/PCBM/C60/BCP/Ag	11.50	22	0.76	0.69	0.60	[87]
ITO/PEDOT:PSS/FASnI_3_/PHCl/C60/BCP/Ag	11.40	23.5	0.76	0.64	0.64	[107]
FTO/PEDOT:PSS/FA_0.98_EDA_0.010_SnI_3_/GeI_2_-<EDA vapor>/BCP/Ag	11.29	24.05	0.66	0.71	0.74	[85]
ITO/PEDOT:PSS/FAPEASnI_3_Br/EABr/PCBM/BCP/Ag	10.80	18.74	0.83	0.69	0.65	[134]
FTO/PEDOT:PSS/FA_0.98_DMA_x_EDA_0.01_SnI_3_-<EDA>/C60/BCP/Ag	10.78	24.30	0.63	0.78	0.80	[135]
FTO/PEDOT:PSS/FASnI_3_/Dipl/NaBH_4_/C60/BCP/Ag	10.61	22.13	0.66	0.73	0.72	[115]
FTO/PEDOT:PSS/FA_0.8_GA_0.2_SnI_3_/C60/BCP/Ag	10.60	21.10	0.645	0.76	0.755	[80]
ITO/PEDOT:PSS/FA_0.95_MA_0.05_EDA_0.01_SnI_3_/PCBM/BCP	10.58	23.16	0.68	0.67	0.71	[129]
PET/ITO/NiOx/FASn_0.9_Ge_0.2_I_3_/4(AMP)I_2_/PCBM/BCP/Ag	10.43	21.3	0.69	0.71	0.71	[77]
ITO/PEDOT:PSS/FASnI_3_/LFA/C60/BCP/Ag	10.37	22.25	0.63	0.74	0.77	[136]
ITO/PEDOT:PSS/FA_0.75_MA_0.25_SnI_3_/PEAI/C60/BCP/Ag	10.30	21.62	0.63	0.76	0.74	[98]
FTO/PEDOT:PSS/(EA_0.1_FA_0.9_)_0.98_EDA_0.010_SnI_3_/GeI_2_-DMF as stirring solvent/BCP/Ag	10.28	23.32	0.59	0.75	0.81	[94]
FTO/PEDOT:PSS/(DEA_x_FA_1–x_)_0.98_EDA_0.01_SnI_3_/GeI_2_/<EDA>/C60/BCP/Ag	10.28	21.69	0.67	0.71	0.75	[86]
ITO/PEDOT:PSS/PEA_0.15_EA_0.15_FA_0.70_SnI_2.7_Br_0.3_/PCBM/BCP/Ag	10.12	21.12	0.68	0.70	0.80	[81]
ITO/FA_0.95_MA_0.05_EDA_0.01_SnI_3_/PCBM/BCP	10.11	22.03	0.67	0.67	0.72	[129]
ITO/PEDOT:PSS/CsSnI_3_-PTM/ICBA/BCP/Ag	10.10	21.81	0.64	0.72	0.67	[137]
ITO/PEDOT:PSS/FASnI_3_/2F-PEAI/EDAI_2_/<DMF-AP> solvent/C60/BCP/Ag	10.03	22.02	0.65	0.71	0.75	[103]

$\mathrm{Voltage}\;\mathrm{loss}\;\left(\mathrm{eV}\right)=E_{g}-{\mathrm{qV}}_{\mathrm{oc}}$; < > denotes the surface passivation.

**Table 6:** The summary of the thermoelectric performance of Sn-PVK films.

Sn-PVK	*σ* [S/cm]	*S* [µV/K]	Power Factor [µW/m·K^2^]	K [W/m·K]	ZT	Measurement Temp	Ref.
CsSnI_3_	124	+115	164	0.36	0.137	27 °C	[141]
MASnI_3_	3.56	+66.03	1.55	-	-	50 °C	[150]
CsSnI_3_/Y_2_O_3_	316.67	+56.08	99.60	0.28	0.11	27 °C	[76]
CsSnI_3_	106	+117	145.10	-	-	27 °C	[38]
CsSnI_3_	115	+113.68	148.61	-	-	27 °C	[40]
CsSnI_3_/Al_2_O_3_	50	+62.63	19.61	-	-	27 °C	[40]
CsSnI_3_/NiO_x_	180	+84.80	127.25	-	-	27 °C	[40]
CsSnI_3_/TiO_2_	130	+81.42	100.94	-	-	27 °C	[40]
CsSnI_3_/ZnO	55	+107.58	63.65	-	-	27 °C	[40]
CsSnI_3_/ZrO_2_	288	+80.49	186.58	-	-	27 °C	[40]
MASnI_3_	1.64	+1.01	1.55	-	-	46 °C	[151]
CsSnI_3_ film	50	+118	-	0.69	0.003		[156]
Cs_2_SnI_6_ film	0.128 ± 0.007	−255 ± 18	-	0.30	0.0011	100 °C	[156]
CsSn_0.8_Ge_0.2_I_3_ bulk crystal	190.1	+92.2	162	0.60	0.088	27 °C	[144]
CsSnI_3_ film	50	110	60	0.28	0.08	-	[143]
CsSnI_3−x_Cl_x_ film	105	115	-	0.38	0.14	345 K	[146]
FASnI_3_/F_4_ATCNQ	18	175	53	0.296	0.05	27 °C	[148]
MASnI_3_	22	78	-	-	-	27 °C	[153]
MAPb_0.75_Sn_0.2_5I_3_	1.2	114	-	-	-	27 °C	[153]
FASnI_3_	2.81	213	12.74	0.141	0.03	-	[149]
FASnI_3_-F4TCNQ	13.65	310	130	0.212	0.19	27 °C	[149]
CsSnI_3_ ingot	65	133	115	0.37	0.15	550 K	[145]

## Data Availability

Not applicable.
